# Functional foods and bioactive compounds: a comprehensive review on their role in mitigating drug-induced liver injury

**DOI:** 10.3389/fnut.2024.1499697

**Published:** 2025-05-21

**Authors:** Jin-Wei Zhao, Wei-Yi Zhao, Meng Zhao, Lu Yu

**Affiliations:** Department of Hepatopancreatobiliary Surgery of Second Hospital of Jilin University, State Key Laboratory for Diagnosis and Treatment of Severe Zoonotic Infectious Diseases, Key Laboratory for Zoonosis Research of the Ministry of Education, Institute of Zoonosis, and College of Veterinary Medicine, Jilin University, Changchun, China

**Keywords:** functional foods, food-derived bioactive ingredients, drug-induced liver injury, protective effects, mechanism

## Abstract

Drug-induced liver injury (DILI) has become a serious public health issue worldwide. Many drugs (chemotherapy drugs, fever-reducing medications, nonsteroidal anti-inflammatory drugs, immunosuppressants, antibiotics, antivirals, and antineoplastic drugs, etc.) may cause liver damage and potentially lead to acute liver failure (ALF). There is an urgent need to develop effective treatment programs for DILI. Here, the epidemiology, pathogenesis and molecular mechanisms of DILI, the reported functional foods and dietary bioactive constituents, such as phenols, flavonoids, glycosides, terpenes, and carotenoids, isolated from food (legumes, nuts, grains, fruits, spices and vegetables, etc.) and their protective mechanisms against DILI are summarized and classified. Research shows that antipyretic and analgesic drugs (such as acetaminophen) are the most common causes of drug-induced liver injury (DILI). Compounds derived from food, particularly flavonoids, have been extensively studied for their ability to alleviate liver damage caused by acetaminophen. They exert significant hepatoprotective effects by preventing mitochondrial dysfunction and oxidative stress, as well as inhibiting inflammation. However, reducing the toxicity of food-derived compounds and improving their solubility and bioavailability in the treatment of drug-induced liver injury remain current and future challenges to address. Future research on and application of anti-DILI dietary bioactive compounds are also needed. Overall, this review may provide insights into the potential use of functional foods and dietary bioactive compounds in the treatment of DILI.

## 1 Introduction

Drug-induced liver injury (DILI) is increasingly significant and contributes to acute liver failure (ALF) and acute hepatitis. In the Western world, acetaminophen (APAP, also known as paracetamol) overdose is the most common cause of drug-induced ALF. However, in Asia, anti-tuberculosis treatment and traditional herbal medicines are major sources of DILI and drug-induced ALF, especially in India and China (1). Many drugs are known to cause DILI, such as chemotherapy drugs, antipyretics, NSAIDs, immunosuppressants, antibiotics, antiviral drugs (2–4), and anticancer drugs (5, 6). Therefore, DILI is considered a serious health issue and has attracted global attention in the fields of toxicology, public health, nutrition, and food science.

For hundreds of years, many foods and edible plants have been believed to have therapeutic effects on disease. Currently, an increasing number of such foods have been successfully developed as functional food products. Many dietary bioactive compounds, such as carotenoids, flavonoids, saponins and terpenes, have preventive and therapeutic effects on DILI due to their strong antioxidant, anti-inflammatory, anti-apoptotic, autophagy-inducing and ferroptosis-inhibiting effects (7). Given the rising global incidence of DILI, identifying natural therapeutic interventions such as functional foods and bioactive compounds has become an urgent area of research. Nevertheless, issues such as drug side effects, low solubility, and bioavailability urgently need to be addressed in food-derived compounds against DILI. This review discusses the reported liver-protective effects of dietary bioactive compounds on DILI, with a particular focus on their potential role in APAP-induced liver injury, in addition, we also discuss the limitations or negative effects of food-derived active ingredients on DILI, as well as possible solutions. We believe this review will provide new insights to further explore food-derived compounds in preventing and managing DILI.

## 2 Epidemiology and etiology of DILI

**Figure 1:**
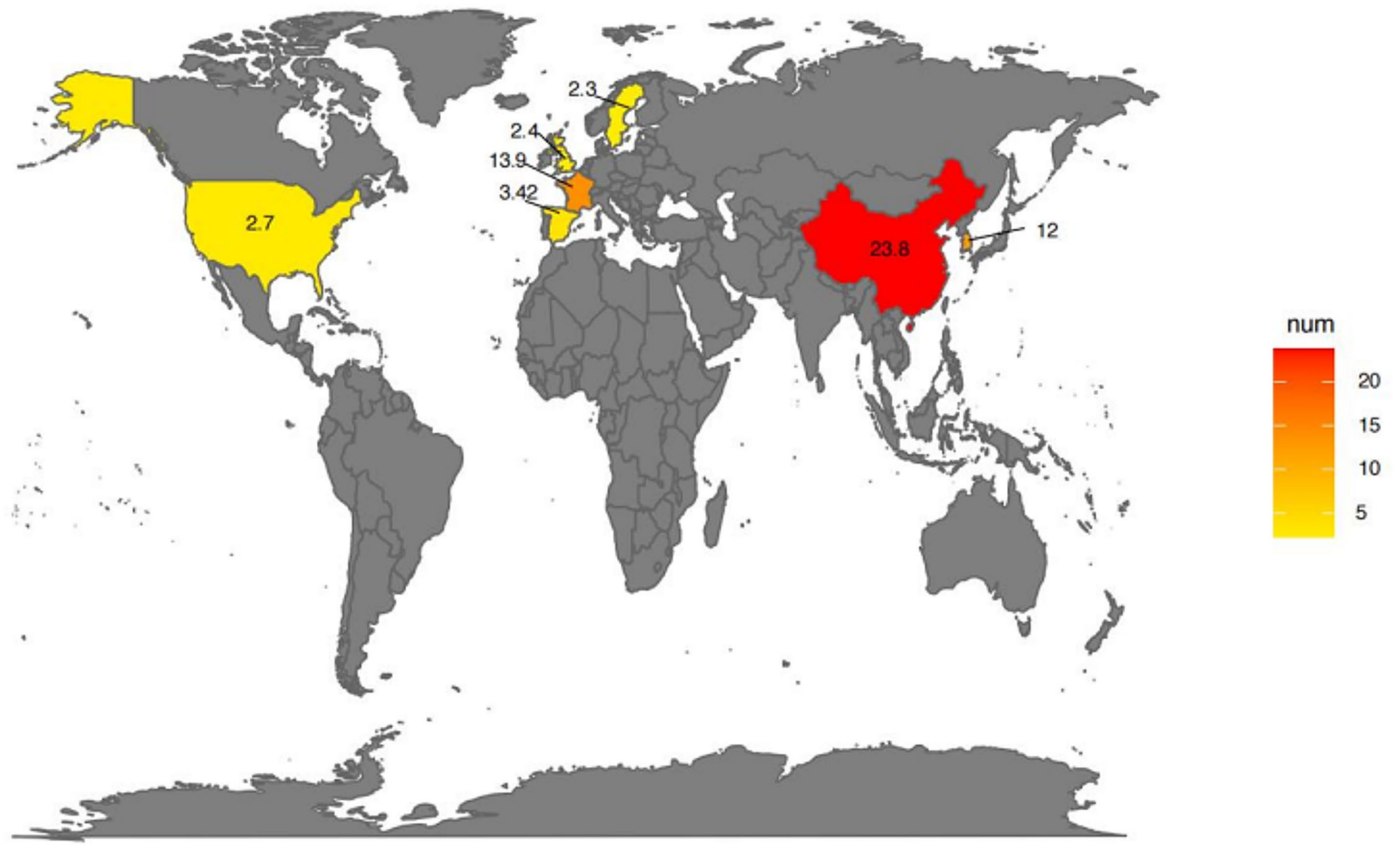
Global prevalence of drug-induced liver injury (DILI). The map gradient color indicates the prevalence of DILI on different continents. Created using SmartDraw.com.

Epidemiological data on DILI have been reported from different countries and regions around the world. The incidence of DILI varies due to factors such as the research cohort and its design, population distribution and immigration status, disease diagnostic criteria, and types of drugs used (Figure 1). The incidence of DILI can be traced back to the epidemiological survey database of the general population research database (GPRD) of the early 1990s. According to published retrospective studies, the incidence of DILI in the UK, Sweden and Spain is approximately 2.4 cases, 2.3 cases and 3.42 cases per 100,000 people annually, respectively (8). However, in Asia, the incidence of DILI in the general population is approximately 12 cases per 100,000 individuals each year in South Korea (9) and in China, with an estimated rate of 23.8 cases per 100,000 individuals (10). In a prospective study based on the French population, the annual incidence rate of DILI was estimated to be 13.9 per 100,000 residents (11). Additionally, a prospective study monitoring approximately 930,000 residents of Delaware in the United States reported an incidence rate of 2.7 per 100,000 adults for DILI (12).

Research has shown that there are significant regional and temporal differences in the pathogenic factors of DILI in Asia, Europe, Latin America, and the United States. APAP is the main cause of DILI in British and American populations, while traditional Chinese medicine and dietary supplements are the main causes of DILI in Asian populations (13). An increasing amount of epidemiologic data, especially from recent cohort studies, have revealed that hepatotoxicity is caused by antineoplastic drugs (5, 6), which include traditional chemotherapeutic agents, tyrosine kinase inhibitors (TKIs), immune checkpoint inhibitors (ICIs), and immunomodulators used for multiple sclerosis and anti-tumor necrosis factor (anti-TNF) drugs (14–22). These drugs are often associated with immune-related adverse events that play a role in mediating hepatotoxicity, representing an important yet understudied category of pharmaceuticals. Overall, while APAP remains the primary cause of DILI in Western populations, traditional medicines and supplements are more prevalent in Asia, underscoring the diverse etiology of DILI globally.

## 3 Pathogenesis and molecular mechanisms of DILI

**Figure 2:**
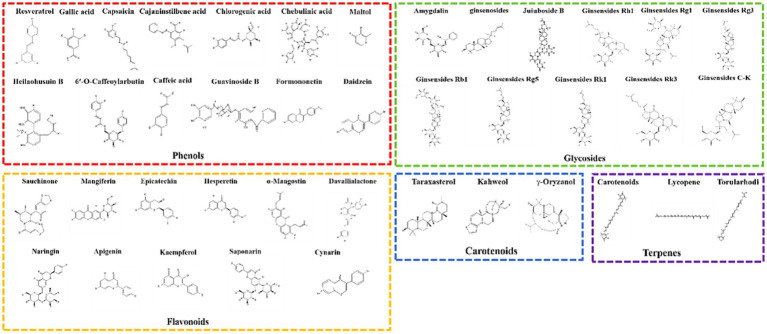
Molecular mechanisms of drug-induced liver injury (DILI). The molecular mechanisms of DILI include: (1) mitochondrial dysfunction; (2) oxidative stress; (3) bile salt export pump (BSEP) inhibition; (4) immune and inflammation response-mediated hepatotoxicity; and (5) apoptosis, autophagy and ferroptosis. Created with BioRender.com.

Drugs that can cause hepatotoxicity include NSAIDs, cancer medications, antituberculosis drugs, antibiotics, antifungal agents and antipsychotic medications. In recent years, detailed research has been conducted on the possible molecular mechanisms of DILI caused by different types of drugs (Figure 2). Among them, the most frequently studied drug that causes intrinsic DILI is APAP (19).

### 3.1 Mitochondrial dysfunction

Mitochondrial dysfunction plays a central role in the hepatotoxic effects of drugs like APAP. Drugs that are metabolized by cytochrome P (CYP enzymes), particularly CYP1A2, CYP2C9 and CYP3A4, are more likely to produce reactive metabolites and lead to liver toxicity. The parent drug transforms into reactive metabolites during phase I metabolism by incorporating specific functional groups that react strongly with proteins, such as hydroxyl, carboxyl, amino, or thiol groups (19). Reactive oxygen species (ROS) and reactive nitrogen species (RNS) are produced when drugs interact with proteins and lipids in cell membranes through oxidative stress (20). Moreover, they can disrupt the cellular redox balance, trigger apoptosis of lymphocyte signaling (21), and cause inflammation by releasing proinflammatory cytokines (22). APAP is metabolized by p450 proteins (primarily CYP2E1 and CYP1A2) to form the reactive metabolite n-acetyl-p-benzoquinone imide (NAPQI), which is rapidly coupled to glutathione (GSH) (23). When hepatic GSH levels are limited, free unconjugated NAPQI reacts with sulfhydryl groups on cysteine and lysine residues to produce NAPQI protein adjuncts (APAP protein adjuncts), which are thought to be key to the onset of hepatotoxicity, leading to oxidative stress, mitochondrial dysfunction, and causing hepatocyte necrosis and inflammation and immune response (13, 21, 22, 24). Due to the mitochondrial dysfunction caused by APAP hepatotoxicity, the removal of damaged mitochondria through mitophagy is an important mechanism for APAP-induced ALF (25).

When mitochondria are significantly stimulated, abnormalities in mitochondrial structure and function can occur in the following ways: morphological and structural changes, abnormal energy metabolism, increased levels of reactive oxygen species (ROS), damage to mitochondrial DNA, and abnormal mitophagy (26–28). The PTEN induced putative kinases 1 (PINK1)/Parkin pathway, activated by phosphatase and tensin homolog (PTEN) genes, regulates functions such as the generation of autophagosomes, mitochondrial division, and fusion with lysosomes during autophagy. In short, in response to various stimuli, PINK1 targets damaged mitochondria from the inner mitochondrial membrane to the outer membrane and then recruits Parkin to eliminate the damaged mitochondria. Importantly, inhibiting PINK1/Parkin increases the hepatotoxicity of APAP by impairing liver autophagy, suggesting that PINK1/Parkin-mediated autophagy may be crucial for reducing APAP toxicity (29, 30). Further research indicates that the dietary compound chlorogenic acid (CGA) promotes the co-localization of translocase of outer mitochondrial membrane 20 (Tom20) and microtubule-associated protein 1 light chain 3 (LC3II) in mitochondria, significantly increasing the levels of genes and proteins associated with mitochondrial autophagy (PINK1, Parkin, LC3II/LC3I), while significantly decreasing the levels of p62 and Tom20. This suggests that CGA may activate PINK1/Parkin-mediated mitochondrial autophagy in APAP-induced liver injury, thereby inhibiting APAP hepatotoxicity (31) (as shown in Figure 2).

### 3.2 Oxidative stress

Mitochondrial dysfunction disrupts energy metabolism, triggers oxidative stress, and subsequently causes hepatocyte toxicity. The liver is an important source of ROS, which can be generated by the ingestion of APAP. When excessive APAP exceeds the phase II reaction pathway, excess NAPQI depletes GSH, disrupting mitochondrial electron transport chain complexes I/II and causing electrons to leak from the ETC to O_2_, forming O_2_^−^ (32). NAPQI interacts with mitochondrial target DNA and proteins, as well as with protein adducts, leading to oxidative stress and mitochondrial dysfunction (24, 25). Excessive APAP generates NAPQI, which also leads to nitration of protein tyrosine residues, inducing the production of peroxynitrite (ONOO^−^) by inducible nitric oxide synthase (iNOS) (33). ONOO^−^ is produced in mitochondria and is detoxified by reacting with GSH. Due to excessive reactions depleting GSH, ONOO^−^ accumulates. The high reactivity and strong oxidative action of ONOO^−^ cause mtDNA damage and the opening of membrane pores (34). Excess ONOO^−^ can also directly react with carbon dioxide to produce peroxynitrite (ONOOCO_2_^−^), which further decomposes to generate ^•^CO_3_^−^ and OH^•^ radicals, which react with metal centers, leading to hepatocyte toxicity (35, 36). Previous studies have shown that t Kaempferol administration downregulated the expression of cytochrome P450 2E1 (CYP2E1) and upregulated the expression of UDP-glucuronosyltransferase family 1 member A1 (UGT1A1), thereby inhibiting the formation of thiobarbituric acid reactive substances (TBARS) and 3-nitrotyrosine (3-NT). This also restores the activities of superoxide dismutase (SOD), glutathione peroxidase (GPx), and catalase to normal levels, maintains normal glutathione levels, and reduces c-Jun N-terminal kinase (JNK) and extracellular regulated protein kinases (ERK) phosphorylation. Kaempferol administration inhibited JNK shifts to the mitochondria and lowered a mitochondrial permeability transition, which reduces mitochondrial oxidative stress and mitochondrial dysfunction that leads to nuclear DNA damage, thereby protecting the liver against propacetamol-induced injury (37) (as shown in Figure 2).

### 3.3 Immune and inflammation response-mediated hepatotoxicity

The immune and inflammation response is also crucial in drug-induced liver injury (DILI). Prolonged exposure to drugs can lead to inflammation and drug-induced autoimmune hepatitis (DIAIH), or acute liver toxicity. The mitochondrial dysfunction triggers cell necrosis; subsequently, necrotic hepatocytes release various endogenous damage-associated molecular patterns (DAMPs) (38). The activation of liver macrophage inflammasomes induced by DAMPs can occur through the toll-like receptor (TLR) pathway (39). When Toll-like receptors (TLRs) are bound by their ligands, they trigger the formation of inflammasomes, leading to transcriptional activation of pro-IL-1β and the release of active IL-1β and IL-18, suggesting that inflammasome activation is the beginning of sterile inflammation (40). The innate immune system is further activated by the release of IL-1β and IL-18, resulting in increased production of proinflammatory cytokines or chemokines (41). This concept is supported by elevated levels of inflammatory factors in plasma in patients who overuse APAP (42, 43) or in laboratory animals who overdose APAP (44, 45).

Furthermore, in the immune response mediated by APAP hepatotoxicity, Kupffer cells form the first line of defense by recognizing DAMPs released from necrotic liver cells. Upon activation, these cells release cytokines such as IL-6, interferon (IFN), tumor necrosis factor (TNF), and chemokines (46). These cytokines recruit and activate neutrophils and monocytes, upregulating adhesion molecules on liver sinusoidal endothelial cells (LSECs) and hepatocytes. By inducing the expression of inflammatory mediators and adhesion molecules, they assist neutrophils in adhering and transmigrating within the sinusoids, adhering to target cells and relying on oxidative stress to regulate the aggregation of immune cells, leading to hepatocyte death (47, 48). Additionally, Gerussi et al. suggested that the host adaptive immune system is primarily influenced by factors such as HLA polymorphisms, which affect the presentation of hapten peptides or novel antigens and play a critical role in the occurrence and development of specific DILI (49) (as shown in Figure 2).

### 3.4 Apoptosis, autophagy and ferroptosis

Programmed cell death, such as apoptosis, autophagy, and ferroptosis, is one of the important mechanisms of drug-induced liver injury. As mentioned above, drugs metabolized by CYP enzymes, especially CYP1A2, CYP2C9, and CYP3A4, are more likely to produce active metabolites and cause liver cell death, mainly through apoptosis, autophagy, and ferroptosis. Apoptosis of liver cells is directly involved in the pathogenesis of liver toxicity. Increasing studies show that acetaminophen (APAP) induces the translocation of B-cell lymphoma-2 (Bcl-2) family proteins, including the upregulation of the pro-apoptotic protein Bax, downregulation of the anti-apoptotic protein Bcl-2, and activation of caspase-3, thereby promoting hepatocyte apoptosis (50–53).

The activation of autophagy may lead to autophagic cell death and regulate apoptotic cell death by modulating Bcl-2 family proteins. Bcl-2 proteins not only reduce apoptosis by countering proapoptotic proteins but also interact with Beclin-1 to hinder autophagy. Proteins like Bcl-2, Bcl-XL, and Bcl-B bind to Beclin-1, blocking its association with the phosphatidylinositol 3-kinase catalytic subunit type 3 (PI3KC3) complex and inhibiting autophagy. BNIP3 (BCL2/adenovirus E1B protein-interacting protein 3) induces apoptosis by sequestering Bcl-2 family proteins, facilitating the release of proapoptotic mediators through Bax/Bad, and disrupting Bcl-2 family proteins interaction with Beclin-1. Bcl-2 family proteins can coordinately regulate autophagy and apoptosis. Additionally, caspases cleave and inactivate Beclin-1 during apoptosis, which may lead to the suppression of autophagy by apoptosis-effector molecules (54).

During the autophagy process, autophagosomes assemble under the influence of stress signals and fuse with lysosomes to form autolysosomes, which degrade carriers such as incorrectly folded proteins and damaged organelles (55). Recent studies have shown that APAP-induced inhibition of autophagy leads to hepatic lipotoxicity and exacerbated inflammatory reactions; using liver-protective drugs can activate autophagy to reduce hepatic lipotoxicity and inflammation while preventing drug-induced liver damage (53, 55–58). Research by Yuan and others found that in the liver tissue of mice injected with APAP, there was an increase in the expression of Bax, BNIP3, and caspase-3 proteins, a significant increase in the LC3 II/LC3 I ratio, and a decrease in Bcl-2 protein expression. Treatment with alpha-mangostin (*α*-MG) reversed these changes, suggesting that the excessive activation of autophagy and apoptosis induced by APAP injection may be suppressed by α-MG treatment (53). This result further confirms that autophagy and apoptosis are jointly regulated by Bcl family proteins.

Furthermore, glutathione peroxidase 4 (Gpx4) is a central regulatory factor in ferroptosis, and Gpx4 is directly or indirectly inhibited when GSH is depleted, leading to lipid peroxidation and eventually inducing ferroptosis (59). Recent studies have shown that there is significant cell death and lipid peroxidation in the livers of mice treated with APAP, accompanied by reduced expression of Gpx4 and decreased levels of GSH. However, the ferroptosis inhibitor Fer-1 significantly alleviated the aforementioned changes induced by APAP. This evidence strongly supports the involvement of ferroptosis in the mechanism of AILI and suggests that ferroptosis may be one cause of AILI (60–64). Cai and colleagues found through *in vitro* and *in vivo* studies that APAP treatment disrupted iron homeostasis, damaged mitochondrial structure, and downregulated gene and protein expression levels of solute Carrier Family 7 Member 11 (SLC7A11), GPX4, ferritin heavy chain 1 (FTH1), and ferritin light polypeptide 1 (FTL1). However, administering the dietary compound astaxanthin (ASX) reversed these changes. Ultimately, these results indicated that while APAP challenge increased ferroptosis, ASX intervention enhanced the ability to resist it intervention enhanced the ability to resist it intervention enhanced the ability to resist it (64) (as shown in Figure 2).

### 3.5 Bile salt export pump (BSEP) inhibition

The inhibition of BSEP results in the accumulation of toxic bile salts in hepatocytes, leading to cholestatic liver cell injury. BSEPs are protein transporters that transport bile acids out of liver cells, playing a crucial role in eliminating drugs from the liver and secreting bile salts into bile. Inhibition of BSEP expression can result in the buildup of cytotoxic bile acids within the liver, which can damage liver cells and possibly progress to cirrhosis (65). Drugs associated with cholestatic and mixed cholestatic-hepatocellular injuries, including cyclosporine, ritonavir, rosiglitazone, saquinavir, troglitazone, ketoconazole, pioglitazone, lovastatin, haloperidol, atorvastatin, cerivastatin, bosentan, and chlorpromazine, demonstrated strong inhibition of BSEP activity (66, 67). This is due to their ability to inhibit liver transporters and induce DILI by inhibiting BSEP. Elevated levels of hepatotoxic drugs in the bloodstream can increase the risk of liver damage, often as a result of organic anion-transporting polypeptide 1B1 (OATP1B1) inhibition by drugs such as cyclosporine A (68), gemfibrozil (68) and tyrosine kinase inhibitors (TKIs) (pazopanib) (69). Inhibited efflux transporters cause the accumulation of toxic metabolites, leading to liver damage (as shown in Figure 2).

## 4 The effects and its molecular mechanisms of functional foods and diet-derived compounds against DILI

**Figure 3:**
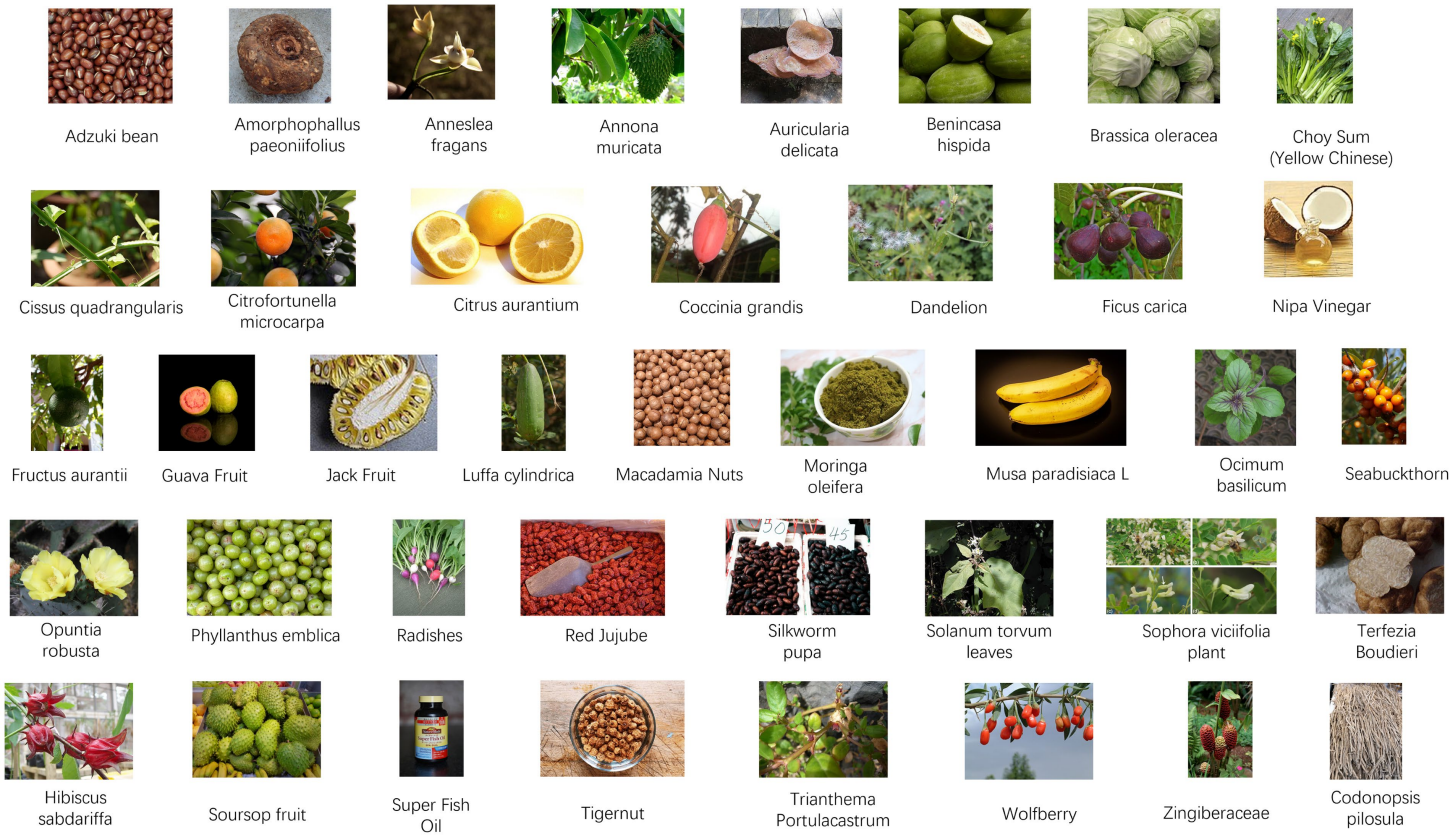
Foods with anti-drug-induced liver injury (DILI) effects. Adzuki bean reproduced by Sanjay Acharya, licensed under CC BY-SA 3.0 via Wikimedia Commons. *Amorphophallus paeoniifolius* reproduced by Aruna, licensed under CC BY-SA 3.0 via Wikimedia Commons. *Anneslea fragrans* reproduced from Tony Rodd, licensed under CC BY-NC-SA 2.0. *Annona muricata* Linn reproduced from Fpalli, licensed under CC BY-SA 3.0 via Wikimedia Commons. *Auricularia delicata* reproduced from Dick Culbert, licensed under CC BY 2.0. *Benincasa hispida* reproduced from Judgefloro, licensed under CC BY-SA 4.0 via Wikimedia Commons. *Brassica oleracea* L. reproduced by Forest & Kim Starr, licensed under CC BY 3.0 via Wikimedia Commons. Choy Sum (Yellow Chinese) reproduced from Anna Frodesiak, licensed under CC0 via Wikimedia Commons. Cissus quadrangularis reproduced from Dinesh Valke, licensed under CC BY-SA 2.0 via Wikimedia Commons. Citrofortunella microcarpa reproduced by David J. Stang, licensed under CC BY-SA 4.0 via Wikimedia Commons. *Citrus aurantium* reproduced from Genet (Diskussion) at German Wikipedia, licensed under CC BY-SA 3.0. Coccinia grandis reproduced by Abdullah AL Shohag, licensed under CC BY-SA 2.0 via Wikimedia Commons. Dandelion reproduced from John Samuel, licensed under CC BY-SA 4.0 via Wikimedia Commons. *Ficus carica* reproduced from H. Zell, licensed under CC BY-SA 3.0 via Wikimedia Commons. Nipa vinegar reproduced from Phu Thinh Co, licensed under CC BY-SA 2.0 via Wikimedia Commons. *Fructus aurantii* reproduced from Ανώνυμος Βικιπαιδιστής, licensed under CC BY 3.0 via Wikimedia Commons. Guava Fruit reproduced from Rodrigo Argenton, licensed under CC BY-SA 4.0 via Wikimedia Commons. Jackfruit reproduced from Alex Popovkin, licensed under CC BY 2.0 via Wikimedia Commons. *Luffa cylindrica* Linn reproduced from Bernand Dupont, licensed under CC BY-SA 2.0 via Wikimedia Commons. Macadamia nut reproduced from Fumikas Sagisavas, licensed under CC0 1.0 via Wikimedia Commons. *Moringa oleifera* reproduced from Satyalatha, licensed under CC BY-SA 4.0 via Wikimedia Commons. *Musa paradisiaca* L. reproduced from Krzysztof Golik, licensed under CC BY-SA 4.0 via Wikimedia Commons. *Ocimum basilicum* reproduced from Rasbak, licensed under CC BY-SA 3.0 via Wikimedia Commons. Seabuckthorn reproduced from Hans Hillewaert, licensed under CC BY-SA 3.0 via Wikimedia Commons. *Opuntia robusta* reproduced from George Hull, licensed under CC BY-SA 2.0 via Wikimedia Commons. *Phyllanthus emblica* reproduced from Ji-Elle, licensed under CC BY-SA 4.0 via Wikimedia Commons. Radishes reproduced Jengod, licensed under CC BY-SA 3.0 via Wikimedia Commons. Red jujube reproduced from Richard, licensed under CC BY 2.0 via Wikimedia Commons. Silkworm pupa reproduced from Photo by 金枫 郭 via Pexels. *Solanum torvum* leaves reproduced from Forest & Kim Starr, licensed under CC BY 3.0 via Wikimedia Commons. *Sophora viciifolia* fruit reproduced from Pan et al. (208), licensed under CC BY 4.0. *Terfezia Boudieri* reproduced from Daniel B. Wheeler, licensed under CC BY-SA 3.0 via Wikimedia Commons. *Hibiscus sabdariffa* L. reproduced by Suresh Aru, licensed under CC BY 2.0 via Wikimedia Commons. Soursop fruit reproduced from Gérard from Nouméa, licensed under CC BY-SA 2.0 via Wikimedia Commons. Super Fish Oil reproduced from Nagato Yuki, licensed under CC BY-SA 4.0 via Wikimedia Commons. Tigernut reproduced from Enrique. garzo. cano, licensed under CC BY-SA 4.0 via Wikimedia Commons. *Trianthema portulacastrum* L. reproduced by Forest and Kim Starr, licensed under CC BY 3.0 via Wikimedia Commons. Wolfberry reproduced from Paul144, Public domain via Wikimedia Commons. *Zingiberaceae* reproduced by Dr. Alexey Yakovlev, licensed under CC BY-SA 2.0 via Wikimedia Commons. *Codonopsis pilosula* reproduced from Elvgashea SIX, licensed under CC0 1.0 VIA Wikimedia Commons.

**Figure 4:**
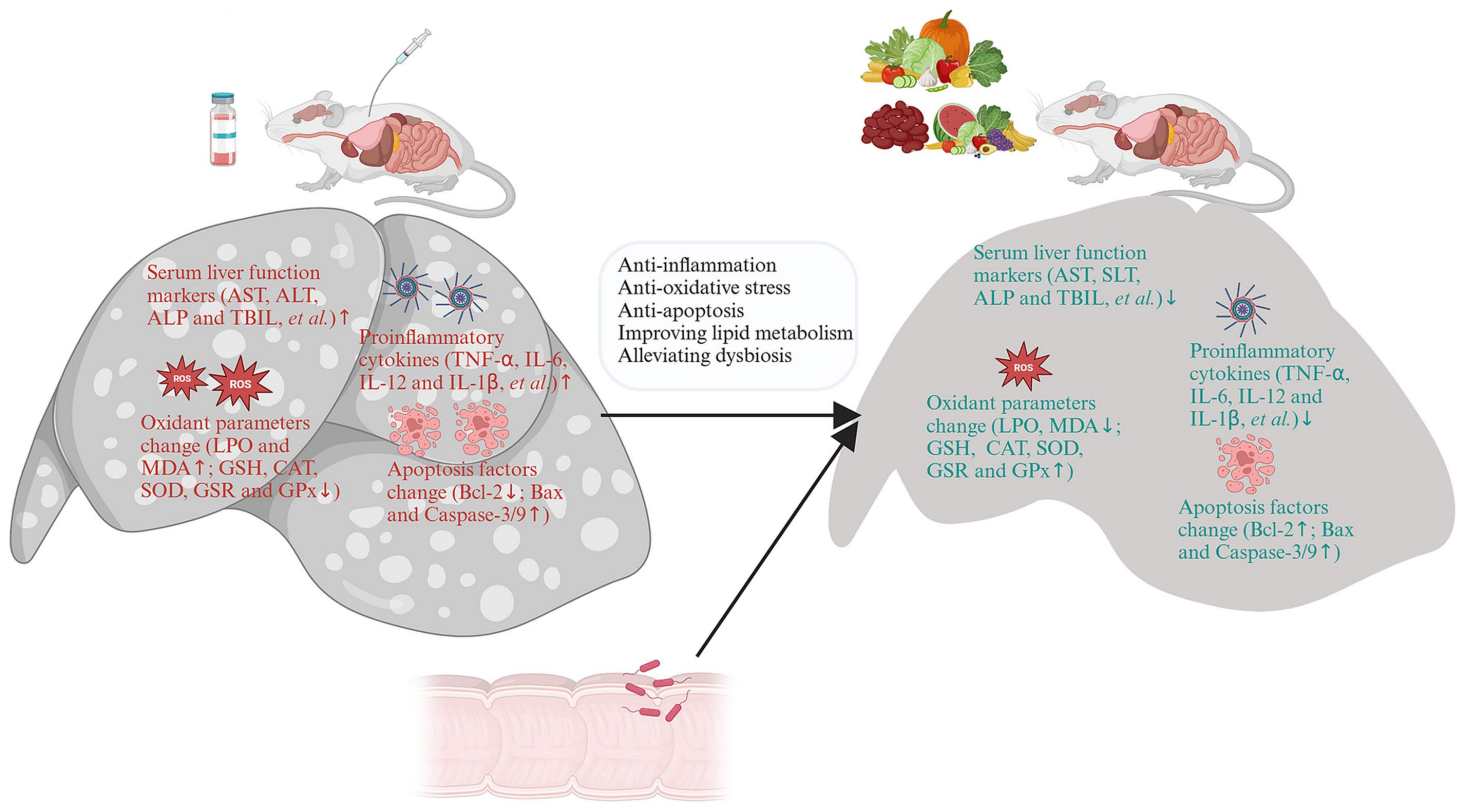
The mechanisms against drug-induced liver injury (DILI) of foods. The molecular mechanisms against DILI include the anti-inflammation, anti-oxidative defense, anti-opoptosis, improve lipid metabolism and alliviate dysbiosis. Created with BioRender.com.

This review includes an assessment of functional foods and their crude extracts reported between 2004 and 2022 for their preventive and therapeutic effects on DILI caused by food, as shown in Figure 3. Additionally, the review isolates bioactive components from food with therapeutic and preventive effects against DILI, mainly including phenolics, flavonoids, glycosides, terpenes, and carotenoids, categorizing other types of food-derived bioactive components within functional foods and crude extracts, as shown in Figure 4.

### 4.1 Foods

**Figure 5:**
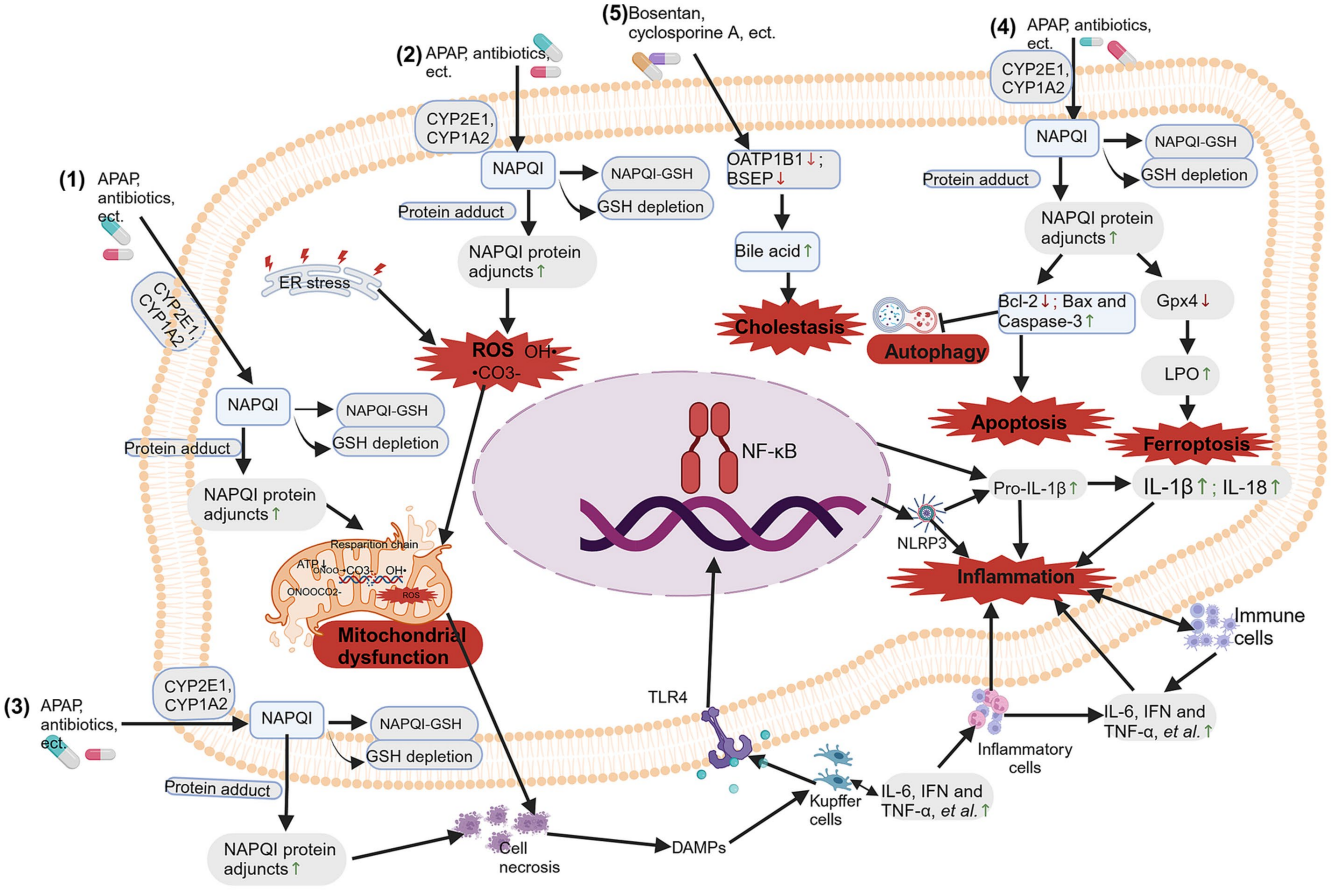
Diet-derived bioactive compounds with anti-drug-induced liver injury (DILI) effects.

Plenty of foods have been widely reported to reduce DILI, with APAP being a prominent example. *In vivo* and *in vitro* experimental animal model studies provided evidence that many kinds of food, such as legumes, seed, fruit, vegetable, spices and oil, have strong hepatoprotective effects, as outlined in Figures 3, 5 and Table 1.

**Table 1:** Foods for the prevention and treatment of drug- induced liver injury.

Bioactive component/extract	Model	Dose (time)	Changes of biological markers	Molecular mechanisms	Authors (Ref.)
Adzuki bean	AAP-induced liver damage in F344/DuCrj rats	5% of diet (4 weeks)	Serum AST↓, hepatic PCOOH and PEOOH↓; hepatic GSH, GSR and CAT↑	Anti-oxidative stress	Han et al. (70)
Sophora viciifolia fruit	APAP-induced liver injury mice	125, 250, 500 mg/kg (7 days)	ALT and AST↓; SOD, CAT, GSH-Px and GSH↑; pathological liver lesions↓; TNF-α, NF-κB and IL-6 mRNA↓; Nrf2, HO-1 and GCLC↑; CYP2E1↓; Nrf2 and Keap1↑	Anti-oxidative stress; anti-inflammation; Keap1-Nrf2 pathway↑	Qi et al. (71)
*Opuntia robusta* and *Opuntia streptacantha*	APAP-induced ALF of Wistar rats	800 mg/kg (5 days)	AST, ALT and ALP↓; liver GSH↑; glycogen↑; leakage of LDH and cell necrosis↓; superior to NAC	Anti-oxidative stress	González-Ponce et al. (72)
Fruits of *Phyllanthus emblica*	Acetaminophen induced hepatic damage in Wister rats	100–200 mg/kg (4 h)	Blood cell count restored; necrosis of hepatocytes↓	Anti-oxidative stress	Malar et al. (73)
*Citrus microcarpa* Bunge/flavonoids, tannins, and glycoside	APAP-induced liver damage in SD rats	4,000 mg/kg (8 days)	ALT, AST and AP levels	Anti-oxidative stress	Franchesca et al. (74)
Musa paradisiacaL.	APAP-induced liver damage in SD rats	10% of diets (60 days)	ALT, AST↓; liver necrosis and regeneration↑	Anti-oxidative stress	Iweala et al. (75)
Musa paradisiacaL.	APAP induced hepatic toxicity in albino rats	200 mg/kg (15 days)	Lipid peroxidation and accumulation of free radicals↓	Anti-oxidative stress	Radhika et al. (76)
*Annona muricata* Linn.	APAP-induced liver damage in SD rats	1 g/kg and 2 g/kg (15 days)	AST, ALT and ALP↓; TP and ALB↑; SOD, GPx, CAT and GHS↑; LPO↓	Anti-oxidative stress	Menon et al. (77)
*Coccinia indica*	Acetaminophen induced hepatotoxicity in rats	200, 400 mg/kg (7 days)	AST, ALT, ALP and TBIL↓, similar to silymarin	Anti-oxidative stress	Sanapala et al. (78)
Red Jujub	APAP-induced liver damage in Wistar rats	70, 140, 280 mg/kg (10 days)	ALT and AST↓	Anti-oxidative stress	Tedyanto et al. (79)
*Lycium barbarum* L. (wolfberry)	APAP-induced DILI in BALB/c mice	0.026 g (5 days)	AST, ALT and TBA↓; YAP1/FXR↑; CYP7A1↓	balance of intestinal microbiota↑; bile acids↓	Lu et al. (80)
*Terfezia Boudieri* (Edible Desert Truffle Specie)/B3 vitamin; quinic acid;chlorogenic acid;quercetin-3-o-rhamonoside	Acetaminophen-induced liver injury in rats	125 mg/kg (4 days)	Necrosis and injury areas, inflammatory cells and Kuppfer cells infiltration in the sinusoid capillaries	Anti-oxidative stress; anti-inflammation	Nouiri et al. (81)
Anneslea fragrans	APAP-induced acute liver injury in mice/HeG2 cells	AFE and AFW (200 or 600 mg/kg)(14 days pre)/(AFW and AFE at 50 and 150 μg/mL) (24 h)	AST and ALT↓; GSH↑; SOD, CAT, HO-1 and NQO-1↑; NO, TNF-α, IL-1β, IL-6 and Bcl-2↓; Bax↑; caspase-3/9↓	Anti-oxidative stress (Nrf2 pathway↑); anti-inflammation (JNK/p38/ERK/NF-κB pathways↓); anti-opoptosis	Hu et al. (82)
*Citrus aurantium L.*	APAP-induced liver injury in Kunming mice/BRL-3A cell	6 g/kg (7 days)/62.5 mM (24 h)	AST and ALT↓; GSH↑; TG↓; P53, BAX and Caspase3↓, p-MKK, p-JNK1 and PUMA↓	anti-opoptosis (AMPK-SIRT1, JNK1 signaling pathways; SIRT1-p53 pathway↓); reversing disorder of liver lipid metabolism	Shu et al. (83)
Tigernut (*Cyperus esculentus* L.)	Acetaminophen-induced hepatotoxicity in rats	500, 1,000, and 2,000 mg/kg (14 days)	GST↑	Anti-oxidative stress	Onuoha et al. (84)
Soursop fruit extract (SSFE)	APAP-induced liver injury in Wistar albino rats	300 mg/kg (7 days)	ALT, AST, ALP and TBIL; MDA, nitrites and nitrates↓; GSH, SOD, CAT, GSR and GPx↑; HO-1; TNF-α, IL-1β↓ and iNOS mRNA↓; Bax and Bcl-2↑, TGF-β↓; release of cytochrome c↓	Nrf2 defense pathway (Nrf2 /HO-1pathway↑); anti-inflammation (NF-κB pathway↓)	Al-Brakati et al. (85)
Macadamia nut protein peptides	APAP-induced liver injury in mice	320 mg/kg and 640 mg/kg (14 days)	ALT, AST and ALP↓; GSH, SOD and GPx↑; HO-1; TLR4, NF-KB, IL-1β and TNF-α gene↓; TNF-α, IL-6↓	Anti-oxidative stress; anti-inflammation; TRL4/NF-κB pathway↓	Shan et al. (86)
Guava Fruit (polysaccharide)	paracetamol -induced liver injury in SD rats	200 g/kg and 400 g/kg (15 days)	ALT, AST and ALP↓; GSH, SOD and GPx↑; TNF-α and IL-6↓	Anti-oxidative stress; anti-inflammation	Alias et al. (87)
Seabuckthorn (polysaccharide)	APAP-induced liver injury in mice	100 mg/kg and 200 mg/kg (30 days)	ALT and AST↓; GSH, SOD, SOD-2 and GPx↑; NO and iNOS↓; JNK phosphorylation↓; Bcl-2/Bax↑; Keap-1↓; Nrf-2↑	Anti-oxidative stress; anti-inflammation; anti-apoptosis; Nrf-2/HO-1-SOD-2 signaling pathway↑	Wang et al. (88)
Cactus cladode extract (*Opuntia ficus-indica*)	MTX-induced liver injury of Wistar rats	0.4 g/kg (10 days)	Hematocrit, hemoglobin and white blood cells↑; biochemical serum parameters↑	Anti-oxidative stress	Akacha et al. (89)
*Hibiscus sabdariffa* calyx/Flavonoid-rich aqueous fraction	Streptozotocin-induced diabetic Wistar rats	1,750 mg/kg (15 days)	GSH, CAT, SOD and GPx↑; AST, ALT and ALP; hepatic fibrosis and glycogen deposition↓	Anti-oxidative stress	Adeyemi et al. (90)
Dried leaves of *Ficus carica*	Rifampicin-Induced Hepatic Damage in Rats	200 mg/100 g (10 days)	SGPT and SGOT↓; cytoarchitecure was restored	Anti-oxidative stress	Gond et al. (91)
Jackfruit (polysaccharides)	Cyclophosphamide-induced liver injury mice	50 mg/kg, 100 mg/kg and 200 mg/kg (7 days)	CAT, SOD and GPx↑; MDA↓; TNF-α, IL-6, IL-2 and IFN-ɣ↓; p-p65/p65 and p-p38/p38↓; κB-α and p-JNK/JNK↑	Anti-oxidative stress; anti-apoptosis; MAPK pathway↓; NF-κB/p65 inflammatory pathway↓	Cheng et al. (92)
*Trianthema portulacastrum* L. (Aizoaceae)	Acetaminophen and thioacetamide induced hepatotoxicity in albino rats	100, 200 mg/kg (10 days)	SGOT, SGPT, ALP and BRN↓; TP↑	Anti-oxidative stress	Kumar et al. (93)
*Moringa oleifera*	Antitubercular drug-induced liver damage in rats	150, 200, 250 mg/kg (45 days)	LPO↓	Anti-oxidative stress	Pari et al. (94)
*Moringa Oleifera* Lam	Acetaminophen-induced liver injury in albino rats	500 mg/kg (7 days)	TBIL, ALT, AST and ALP↓; ALB↑	Anti-oxidative stress	Selim et al. (95)
*Luffa cylindrica* Linn	Isoniazid + rifampicin induced hepatotoxic in rats model	300, 350 mg/kg (21 days)	TP↑; ALP, AST, ALT, TBIL and γ-GT↓; hepatocytic necrosis and inflammation↓	Anti-oxidative stress; anti-inflammation	Pal et al. (96)
*Amorphophallus paeoniifolius* tubers/carbohydrates, proteins, steroids and flavonoids	Acetaminophen induced liver damage in rats	300 mg/kg (4 days)	GOT, GPT, ALP and TBIL↓	Anti-oxidative stress	Hurkadale et al. (97)
Pulps of *Benincasa hispida*	Nimesulide-induced hepatotoxicity model in rats	50 mg/kg (14 days)	GOP, GPT and ALP↓; SOD and CAT↑; GSH and LPO↓	Anti-oxidative stress	Das et al. (98)
Bras sica oleracea L./obtucarbamate, N-(4-hydroxy phenyl) acetamide, and p-hydroxy benzoic acid	Acetaminophen-induced liver injury in rat	1 g/kg (7 days)	GPT and sGOT↓; damaged structural integrity of the liver↓	Anti-oxidative stress	Hashem et al. (99)
Yellow Chinese chive extract (YCE)	APAP-induced hepatotoxicity in mice	25, 100 mg/kg (7 days)	HO-1, NQO1, GPx and xCT expression↑	Anti-oxidative stress (Nrf2 pathway↑)	Kawakami et al. (100)
Dandelion	APAP-induced liver injury in rats	1 g/kg (14 days)	AST, ALT, LDH and ROS↓; GSH and GPx↑; TNF -α, COX-2, CYP2E1, MAPK, JNK and NF-KB p65↓	Anti-oxidative stress; anti-apoptosis; anti-inflammation; MAPK and NF-κB pathways↓	Wang et al. (101)
Dandelion	APAP-induced liver injury in Kunming mice	(120, 250, 500, 1,000 g/120 mL) × 0.5 mL/10 g; (7 days)	ALT, AST, AKP, TNF-α and IL-1β↓; MDA↓; GSH and SOD↑; COX-2 and iNOS↓; Bax, caspase-9 and JNK protein↓; hepatocytenecrosis, inflammatory cell infiltration and congestion	Anti-oxidative stress; anti-apoptosis; anti-inflammation; JNK pathway↓; Nrf-2/HO-1 pathway↑	Zheng et al. (102)
Dandelion (polysaccharides)	APAP-induced liver injury in mice	200, 100, 50 mg/kg, 3 times daily for 2 days	AST, MDA and ROS↓; GSH, SOD and CAT, GPX↑; Nrf2, HO-1 and NQO1↑	Anti-oxidative stress; Nrf2-Keap1 pathway↑	Cai et al. (103)
Sweet basil (*Ocimum basilicum* L.)/phenolic, flavonoid	Acetaminophen-induced liver injury in Wistar rats	200 mg/kg (7 days)	Antioxidant enzymes↑; LPO↓; serum transferase enzymes↓	Anti-oxidative stress	Branislava et al. (104)
Auricularia delicata	Acetaminophen-induced hepatic injury in rats	150 mg/kg (5 days)	Mitochondrial-targeted antioxidant effect of chlorogenic acid	Anti-oxidative stress	Wangkheirak et al. (105)
Radishes (RJ) and turnips (RG)	APAP-induced liver-damaged mice	500, 1,000 mg/kg RJ; 500, 1,000 mg/kg RG; (4 weeks)	Inflammation cell infiltration↓; ALT, AST and MDA↓; GHS, SOD and CAT↑; Nrf-2 and HO-1↑; BAX↓ and BCL-2↑	Anti-oxidative stress; anti-apoptosis	Hwang et al. (106)
Zinglber officinale L. (Zingiberaceae)/6-gingerol, 8-gingerol and zingerone	Adriacycin-induced liver injury in wistar albio rats	24 mg (3 times/week × 6 weeks)	AST, ALT and MDA↓; SOD↑; leucocytes infiltration, cytoplasmic vasculization and fat infiltration↓	Anti-oxidative stress; anti-inflammation	Sakr et al. (107)
Zinglber officinale L. (Zingiberaceae)/6-gingerol, 8-gingerol and zingerone, vitamin E	APAP-induced liver injury in wistar albio rats	Ginger 100 mg/kg; Vitamine E 75 mg/kg for 14 days	AST, ALT, TBIL, Arginase, LPO and MDA↓; SOD↑; TAGs↑	Anti-oxidative stress	Abdel-Azeem et al. (108)
*Solanum torvum* leaves/phenolic fraction	APAP- induced liver injury in C57BL/6 mice	600 and 1,200 mg/kg (18 h)	ALT and AST↓; GSH↑; TBARs↑	Anti-oxidative stress	de Souza et al. (109)
*Codonopsis pilosula* (polysaccharides)	Sterigmatocystin-induced liver injury in mice	300 mg/kg, for 14 days	ALT, AST and LDH↓; TNF-α, IL-1β, IL-6, CCL2 and CCL5↓; MPO↓; SOD, CAT and GPx↑; BcL2↑; Bax↓; Caspase-3↓; gut microbiota dysbiosis↑	Anti-oxidative stress; anti-inflammation; anti-apoptosis; regulation of gut microbiota	Nie et al. (110)
Pineapple vinega/phenolic, phenolic acid	Acetaminophen-induced liver damage in BALB/c mice	0.08 and 2 mL/kg (14 days)	AST, ALT, ALP and TG↓; GSH, SOD, LPO and FRAP↑; iNOS, NO and NF-kB; cytochrome P450↓	Anti-oxidative stress; anti-inflammation	Mohamad et al. (111)
Nipa vinegar/gallic acid, protocatechuic acid and 4-hydroxybenzoic acid	Acetaminophen-induced liver damage in mice	0.08, 2 mL/kg (14 days)	AST, ALT and ALP↓; cytochrome P450 2E1↓	Anti-oxidative stress; anti-inflammation	Beh et al. (112)
Rice derived peptide	APAP-induced liver injury in mice	100, 500 mg/kg (7 days)	ALT, AST and LDH↓; GSH↑; histological and centrilobular necrosis↓	Anti-oxidative stress	Kayoko et al. (113)
Eel oil	Acetaminophen-induced liver injury in Wistar rats	2,000, 4,000 mg/kg (14 days)	GPT, TBIL and MDA↓; GSH↑	Anti-oxidative stress	Heru et al. (114)
Silkworm pupa oil (SPO)	APAP-induced liver injury in Kunming mice	3.75, 7.50 mL/kg (14 days)	ALT and AST↓; TNF-α, IL-6 and IL-12↓; NF-κB p65↓; IκB-α↑; MDA; SOD and GSH-Px↑	Anti-oxidative stress; anti-inflammation; NF-κB signaling pathway↓	Long et al. (115)

#### 4.1.1 Legumes and seeds

The water-extract from adzuki bean (*Vigna angularis*) hulls (70) and *Sophora viciifolia* fruit (71) was found to attenuate cetaminophen (APAP)-induced damage in rat liver by decreasing serum aspartate aminotransferase (AST) activity, restore hepatic glutathione (GSH) content and hepatic glutathione reductase (GSR) and catalase (CAT) activities, exert antioxidant defense (70, 71).

In APAP-induced liver injury of rat or mice model, fruit like *Opuntia robusta* and *Opuntia streptacantha* (72), fruits of *Phyllanthus emblica* (73), *Citrus microcarpa Bunge* (flavonoids, tannins, and glycoside) (74), *Musa paradisiaca L.* (75, 76), *Annona muricata Linn.* (77), *Coccinia indica* (78), Red Jujub (79), *Lycium barbarum L.* (wolfberry) (80), *Terfezia* Boudieri (B3 vitamin; quinic acid; chlorogenic acid; quercetin-3-o-rhamonoside) (81), *Anneslea* fragrans (82), *Citrus aurantium* L. (83), Tigernut (*Cyperus esculentus* L.) (84), soursop fruit extract (SSFE) (85), Macadamia Nut (Protein Peptides) (86), Guava Fruit (Polysaccharide) (87) and Seabuckthorn Berry (Polysaccharide) (88) showed hepatoprotective activity against APAP-induced liver damage. The hepatoprotection effect offered by these foods was reflected by the significant decrease in serum liver function markers levels, such as AST, ALT, alkaline phosphatase (ALP), total bilirubin (TBIL) and reduction of any gross morphological injury to the rat’s liver. Importantly, treatment of rats or mice with fruits ameliorated and restored cellular antioxidant status and oxidative stress (OS)-antioxidant parameters, such as decreased lipid peroxidation (LPO), inhibited malonaldehyde (MDA), restored GSH, increased CAT, Superoxide Dismutase (SOD), and GSR, and glutathione peroxidase (GPx) levels (72–88). In addition, ethanol-aqueous (AFE) and hot-water (AFW) extracts from *Anneslea fragrans* exerts activated nuclear factor erythroid 2-related factor 2 (Nrf2) pathway to increase the hepatic antioxidant properties (82). AFE and AFW extracts exerted anti-inflammation role by suppressing the JNK/p38/ERK/NF-κB pathways (82). Moreover, AFE and AFW extracts alleviated apoptosis via regulating Bcl-2, Bax, and caspase-3/9 protein expressions (82). Furthermore, *fructus aurantii* can prevent APAP-induced liver injury by regulating glycerophospholipid metabolism, fatty acid synthesis, and glycerolipid metabolism (83). In addition, *fructus aurantii* exhibits hepatoprotective effects against APAP-induced liver necrosis by inhibiting PUMA and reversing hepatic lipid metabolism disorders (83). In addition, soursop fruit extract (SSFE) pretreatment alleviated liver injury through regulation of hepatic Nrf2/HO-1 (Heme Oxygenase 1) and downregulation of NF-κB and transforming growth factor-*β* (TGF-β) (85).

Literature reports that excessive exposure to acetaminophen (APAP) inactivates endogenous antioxidants, stimulates ROS, alters mitochondrial permeability, and depletes ATP, ultimately leading to liver damage. Nrf2 is a transcription factor that can bind to the antioxidant response element (ARE), regulating the expression of various intracellular antioxidants and detoxifying molecules, including HO-1. Overexpression of HO-1 has been reported to be associated with increased Fe2+ release and exacerbated iron-mediated ROS production. High doses of APAP may activate the NF-κB pathway by enhancing ROS production. NF-κB is considered the main transcription factor promoting the expression of pro-inflammatory cytokines and other mediators in inflammatory and oxidative responses. The APAP-induced liver injury rat model established by Ashraf Y et al. has confirmed that the expression of Nrf2, HO-1, NF-κB, iNOS, TNF-*α* and IL-1*β* and hepatic TGF-β were increased in the liver of APAP-intoxicated rats (85). However, using SSFE pretreatment, the levels of oxidants (MDA and nitrate/nitrite) were reduced, GSH content was increased, and the activities of antioxidant defense enzymes (SOD, CAT, GSR, and GPx) were activated. In addition, SSFE pretreatment regulated the expression of HO-1 and activated Nrf2 in liver tissue. Meanwhile, SSFE pretreatment led to a decrease in levels of TNF-α and IL-1β, as well as downregulation of iNOS and NF-κB expression. Furthermore, SSFE pretreatment could downregulate the abnormal expression of TGF-β induced by APAP, demonstrating significant protective effects against APAP toxicity (85).

Macadamia nut protein peptides, with Glu, Arg, Asp, Leu, Tyr, and Gly being the major amino acids, alleviated AILI in mice by inhibiting TLR4/NF-κB pathway-related gene (TLR4, NF-κB, IL-1β and TNF-α) activation (86). Furthermore, The protective effects of Seabuckthorn Berry polysaccharide extracts are associated with the activation of the Nrf-2/HO-1-SOD-2 signaling pathway (88).

#### 4.1.2 Fruit

In other DILI rat models, through antioxidant activity mechanisms, fruit like *cactus* cladode extract (*Opuntia ficus-indica*) (89) alleviated MTX (methotrexate)-induced liver damage; *hibiscus sabdariffa* calyx/flavonoid-rich aqueous fraction inhibited chlorpyrifos-induced liver injury (90). Additionally, in the rifampicin-induced rat liver injury model, dried leaves of *ficus carica* improved rifampicin-induced hepatotoxicity and exerted antioxidative defense functions (91). *Artocarpus heterophyllus* Lam. (jackfruit) polysaccharides (JFP-Ps) can protect against cyclophosphamide (Cp)-induced liver injury. Furthermore, JFP-Ps modulated immune responses through the mitogen-activated protein kinase (MAPK)/NF-κB pathway associated with inflammation and cell apoptosis. Metabolomics results indicate that the hepatoprotective effects of JFP-Ps are mainly related to tRNA biosynthesis, sphingolipid metabolism, purine metabolism, and the citric acid cycle (92).

#### 4.1.3 Vegetables

Vegetables such as *Trianthema portulacastrum* L. (Aizoaceae) (93), *Moringa oleifera* (94, 95), *Luffa cylindrica* Linn (96), *Amorphophallus paeoniifolius* tubers (97), pulps of *Benincasa hispida* (98), *Bras sica oleracea* L. (99), yellow Chinese (100), dandelion (101–103), sweet basil (*Ocimum basilicum L.*) (104), *Auricularia delicata* (105), radishes (RJ) and turnips (RG) (106), *Zinglber officinale* L. (*Zingiberaceae*) (107, 108), *solanum torvum* leaves (109), *Codonopsis pilosula* (polysaccharides) (110) were reported to enhance hepatic antioxidant activity through the modulation of antioxidant-mediated mechanism by altering serum antioxidases activities and reduced GSH and LPO levels. Furthermore, research has found that yellow Chinese chive extract (YCE) can enhance the expression of Nrf2 and its target antioxidant enzymes in the liver of mice, including NAD(P)H quinone oxidoreductase 1 (NQO1), glutathione peroxidase (GPx), cystine/glutamate transporter (xCT), especially heme oxygenase-1 (HO-1). Vegetables like dandelion suppressed mitogen-activated protein kinase (MAPK) and NF-κB pathways, inhibited activation of JNK pathway and activating the Nrf-2/HO-1 pathway to inhibit the occurrence of oxidative stress, inflammatory response and apoptosis (101, 102); polysaccharides from dandelion root (DRP) increased the Nrf2 and Keap1 and showed to have a protective effect against liver injury by activation of the Keap1-Nrf2 pathway (103). *Phellinus linteus* (polysaccharides) alleviates oxidative stress by activating the Nrf2 signaling pathway and inducing autophagy to protect against APAP-induced acute liver injury in mice. *Luffa cylindrica* Linn (91), dandelion (100), *Zinglber officinale* L. (107, 108) were found to inhibit inflammatory response through regulating cytokines level in serum and liver tissue, and liver inflammatory cell inflammation. In addition, gut microbiota, may also serve as a mechanism for *Codonopsis pilosula* (polysaccharides) (110) mediated remission of liver injury.

#### 4.1.4 Beverages

For beverages, Pineapple vinegar (total phenolic content and gallic acid as the main functional components) was demonstrated to enhance antioxidant defense and suppress LPO and reduced the expressions of iNOS, NF-κB and the level of NO, and downregulated liver cytochrome P450 protein expression (111). Nipa vinegar, which is rich in polyphenolic acids, was found to contribute to anti-oxidation, anti-inflammation and liver protection effects in paracetamol treated mice (112). In addition, the leaves of *Lithocarpus polystachyus* Rehd. protected liver against APAP-induced hepatotoxicity by inhibiting the PI3K/Akt (protein kinase B) mediated apoptosis signal pathway and inhibiting the NF-κB-mediated signaling pathway (113).

#### 4.1.5 Spices

For spices, it was reported that Eel oil could activate Nrf2 and exert antioxidant defense and hepatoprotective activity by inhibiting SGPT, total bilirubin, MDA, and increasing GSH levels in rats (114). Moreover, Silkworm pupa oil attenuated hepatic injury induced by APAP, which attributed to the suppression of oxidative stress-mediated NF-κB signaling and decreased in proinflammatory cytokines, including TNF-*α*, IL-6, and IL-12 (115).

### 4.2 Food-derived products for the treatment of DILI

**Figure 6:**
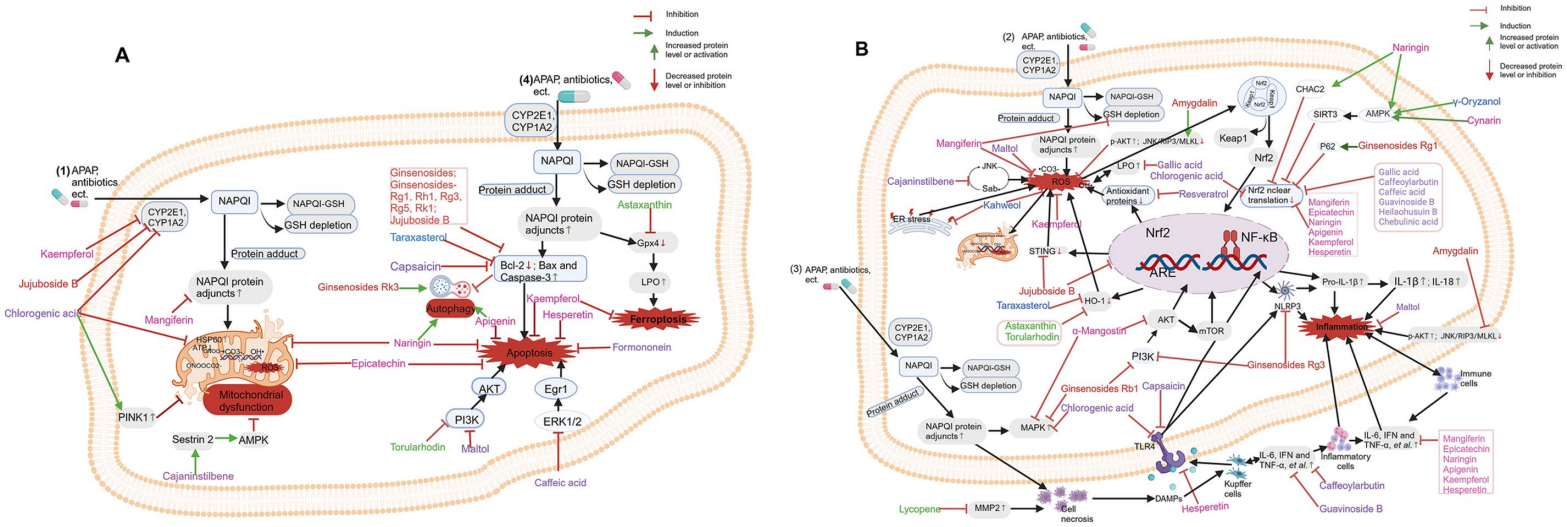
Diet-derived bioactive components and its mechanisms against drug-induced liver injury (DILI). **(A)** Mechanisms of Diet-derived active components in treating DILI: anti-apoptosis, promoting autophagy, and improving mitochondrial dysfunction. **(B)** Mechanisms of Diet-derived active components in treating DILI: anti-opoptosis and anti-inflammation. Phenols in purple; Flavonoids in pink; Glycosides in red; Terpenes in orange and Carotenoids in green. Created with BioRender.com.

A large number of food-derived products have been widely reported to reduce DILI, especially APAP-induced DILI (Figure 4). *In vitro* experiments and *in vivo* animal model studies have demonstrated that a single component of food-derived products, especially phenols, flavonoids, glycosides, terpenes and carotenoids, may be beneficial for the treatment of DILI (Table 2 and Figures 6A,B).

**Table 2:** Diet-derived bioactive compounds for the prevention and treatment of drug-induced liver injury.

Type	Monomers	Diets sources	Model	Does (time)	Changes of biological markers	Molecular mechanisms	Authors (Ref.)
Phenols	Resveratrol	Wine	As_2_O_3_-induced liver injury in cats	3 mL/kg (5 days)	Tissue ROS, GSH/GSSG↓; MDA↓; LPO↓; SOD, GPX and CAT↑; serum TBIL, CHE, AST and ALT↓; cytoplasmic vacuolization, focal necrosis and inflammatory cell infiltration↓; liver As_2_O_3_ concentration↓; LPO↓	Anti-oxidative stress; anti-inflammation	Zhang et al. (116)
	Resveratrol	Grapes and peanuts	In APAP-induced liver damage in C56BL/6 mice	10 mg/kg/d (60 days)	ALB↑; AST↓; Ck18-, F4/80^+^ cells and α-SMA^+^ cells was normalized; BCRP↓	Maintain of mitochondria function;	de Moraes et al. (117)
	Gallic acid	Berberry, pomegranates and gall nuts.	APAP-induced liver injury in Swiss albino mice	100 mg/ kg (4 h)	AST, ALT, ALP, TNF-α and LPO↓; SOD, CAT, GPx, GHR, GST and GSH↑	Antioxidant defense; anti-inflammatation	Rasool et al. (118)
	Gallic acid	Berberry,pomegranates and gall nuts.	Isoniazid and rifampicin-induced liver injury in Wistar Rats	50, 100 and 150 mg/kg (28 days)	Liver function enzymes↓; hepatic necrosis and inflammation↓; Nrf2↑	Anti-oxidant defense; anti-inflammation; inhibited NF-κB/TLR-4 axis↓	Sanjay et al. (119)
	Capsaicin	Chili peppers	APAP-induced liver injury in mice	1 mg/kg (3 days)	MDA↓; SOD and GSH↑; IL-6, IL-1β and TNF-α↓; BCL-2X, caspase-3 and cleaved caspase-3↓; BCL-2↓	Anti-inflammation; anti-oxidant defense; anti-apoptosis; HMGB1/TLR4/NF-κB signaling pathway↓	Zhan et al. (123)
	Maltol	Ginseng	APAP-induced liver injury in ICR mice	50 and 100 mg/kg (7 days)	ALT and AST↓; GSH and SOD↑; MDA↓; CYP2E1 and 4-HNE↓; inflammatory infiltration and apoptosis↓; Bax↓, Bcl-2↑; TNF-α and IL-1β↓	Anti-inflammation;anti-oxidative stress; and anti-apoptosis; NF-κB pathway↓; PI3K/Akt signaling pathway↓	Wang et al. (127)
	6’-*O*-Caffeoylarbutin	Que Zui tea (QT)	APAP-induced liver injury in Kunming mice/HepG-2 cells	20 and 60 mg/kg (7 days); 20, 100 μM (24 h)	Intracellular ROS↓; cell apoptosis↓; SOD, CAT and GSH↑	Anti-inflammation;anti-oxidative stress; and anti-apoptosis	Wang et al. (128)
	Caffeic acid	Coffee	APAP-induced liver injury in ICR mice; liver L-02 cells, HepG2 cells	10 and 30 mg/kg (7 days)/25 and 50 μM (48 h)	ALT/AST↓; MPO and GSH↑; ROS↓; cell viability↑; Nrf2↑; HO-1and NAD(P)H: NQO1↑; Keap1↓; binding of Keap1 to Nrf2↓	Anti-oxidative stress	Pang et al. (130)
	Caffeic acid	Coffee	APAP-induced liver injury in ICR mice	10 and 30 mg/kg (5 h)	Nrf2↑; HO-1↑; NAD(P)H:NQO1↑; PP2A-A and PP5↓; ERK1/2 phosphorylation↑	Anti-oxidative stress; Nrf2 antioxidative signaling pathway↑	Pang et al. (131)
	Cajaninstilbene acid	Pigeon pea	APAP-induced liver injury in C57BL/6 N mice	50 and 75 mg/kg (24 h)	ALT and AST↓; necrotic and apoptotic hepatocytes↓; PGC-1α, TFAM, LC3-II, PINK1 and mitochondrial Parkin↑; p62↓; AMPK; Sestrin2↑	Improve mitochondrial quality control; anti-oxidative stress; Sestrin2/AMPK signaling pathway↑	Yan et al. (132)
	Chlorogenic acid	Fruits, dietary vegetables	APAP-induced liver injury in ICR mice/HepG2 cells	20, 40 mg/kg (14 days)	ALT, AST and LDH↓; cell viability↑; PINK1, Parkin and LC3II/LC3I↑; p62 and Tom20↓	Activate mitophagy, anti-apoptosis. PINK1/Parkin signaling pathway↑	Hu et al. (31)
	Genistein	Soybeans	APAP-induced liver injury in mice	50, 100 and 200 mg/kg (4 h)	ALT, AST, LDH and MDA↓; GSH↑; UGTs and GPx↑; CYP2E↓	Activities of metabolism;antioxidant defense	Fan et al. (134)
	Soy isoflavones	Soybeans	APAP-induced liver injury in rats	120 mg/kg (2 weeks)	ALT↓; GSH↑; CYP2E1 and CYP3A↓	Activities of metabolism and anti-oxidant defense	Liu et al. (135)
	Polyphenols	Tofu	APAP-induced liver injury in albino rats	20 g/day (14 days)	ALP, ALT, AST and LDH↓; TCH and TBIL↓; TP and ALB↑	Anti-oxidant defense	Yakubu et al. (136)
	Guavinoside B	*Psidium guajava*	APAP-induced liver injury in C57BL/6 mice/HepG2 cells	100 mg/kg (7 days)/30 μM (24 h)	Intracellular ROS↓; hepatocyte infiltration and necrosis↓; ALT, AST, ROS, MDA and TNF-α↓; SOD and GSH↑; Nrf2, GCLC and NQO1↑; p-JNK gene expression↓	Anti-oxidative stress; anti-inflammation; Nrf2 and JNK signaling pathways↑	Li et al. (137)
	Formononetin	Legume	APAP-induced liver injury in BALB/c mice/LO2 cells	50 mg/ kg(7 days)/20 and 40 μM (6 h)	AST and ALT↓; apoptotic cytes↓; inflammatory infiltration↓; Nrf2 protein↑; Nrf2 and antioxidant genes mRNA↑	Anti-oxidative stress; anti-inflammation;anti-apoptosis	Jin et al. (138)
	Heilaohusuin B	Kadsura coccinea	APAP-induced HepG-2 cells	5, 10 and 20 μM (18 h)	Nrf2 and HO-1↑	Oxidative stress inhibition via activating the Nrf2 pathway	Yang et al. (139)
Dflavonois	Cynarin	Illyrian thistle	APAP-induced liver injury in mice	25 m g/kg (2 h)	AST and ALT↓; NQO1, HO-1 and Nrf2↑; MDA↓; GPX4↓	Anti-oxidant defense; AMPK/SIRT3 pathway↑	Zhao et al. (142)
	Epicatechin	Grape, cola nuts, straw berries and red wine	STZ-induced diabetes in rats	15 and 30 mg/kg (35 days)	GSH↑; CAT, SOD and GPx↑	Anti-oxidative stress	Quine et al. (144)
	Epicatechin	Grape, cola nuts, straw berries and red wine	MTX-induced diabetes in rats	25, 50 and 100 mg/kg(10 days)	ALT, AST, MDA, IL-1β, TNF-α and NO↓; GSH, CAT, SOD and GHx↑	Anti-oxidative stress	Azadnasab et al. (145)
	Epigallocatechin-3-Gallate	Green tea	APAP-induced liver injury in rats	153 and 460 mg/kg (4 weeks)	CYP1A2, CYP2E1, CYP3A and UGT↓; AST and ALT↓; Bax/Bcl2 ratio↓; LC3B II/I ratio↑; GPx and NQO1↑; OATP1A1↓	Anti-oxidant defense; anti-opoptosis and enhanced autopsy	Yao et al. (147)
	Mangiferin	Mango	APAP-induced liver injury in C57BL/6 mice	25 mg/kg (24 h)	GSH↑; APAP-Cys adduct↓; p-JNK↓; AMPK↑; SOD↑; LPO↓; TNF-α, IL-6, MCP-1, CXCL-1 and CXCL-2 mRNA↓; mRNA and serum levels of IL-1β↓	Anti-oxidative stress; anti-inflammation via JNK pathway	Chowdhury et al. (40)
	Naringin	Grapefruit (*C. paradisi*), *C. aurantium*, and *C. maxima*	MTX-induced liver injury in rats; HepG2 cells	20, 40 and 80 mg/kg (2 weeks); 0.3 mM (48 h)	Serum ALT, AST, ALP and TBIL↓; liver MDA, NO↓, SOD, CAT, GPx, GR and GSH↑; hepatic IL-6 and TNF-α↓	Anti-oxidative stress; anti-inflammation	Elsawy et al. (148)
	Naringin (NG)	Grapefruit (*C. paradisi*), *C. aurantium*, and *C. maxima*	CP-induced liver injury in rats	50 and 100 mg/kg (7 days)	Serum ALT, AST and ALP; SOD, GPx, CAT and GSH↑; MDA↓; TNF-α, NF-κB, IL-6, IL-1α, iNOS and COX-2↓; caspase-3 and LC3B↓; 8-OHdG↓	Anti-oxidant defense;anti-inflammation, anti-apoptosis; promote autophagy	Caglayan et al. (149)
	Naringin	Grapefruit (*C. paradisi*), *C. aurantium*, and *C. maxima*	CP-induced liver injury in rats	50, 100 and 200 mg/kg (14 days)	Serum and hepatic ALT, AST, GGT, ALP and LDH↓; liver MDA, HPx and NO↓; GHS, CAT, GSH, GSH-Px and GSR↑; CCL2, IFN-α1, IL-1β, IL-1R and TGF-β1↓	Antioxidant defense; anti-inflammation	Akamo et al. (150)
	Naringin	Grapefruit (*C. paradisi*), *C. aurantium*, and *C. maxima*	Doxorubicin-induced liver injury in BALB/c mice and AML-12 cells	30, 60, 120 mg/kg (1 weeks); 50, 100 and 200 μM (24 h)	AST, ALT and MDA↓; SOD, GHS and CAT↑; SIRT1↓	Anti-oxidative stress; anti-inflammation; anti-apoptosis	Xi et al. (151)
	Naringin	Grapefruit	Taxol-induced liver injury in Wistar rats	10 mg/kg (every other day for 6 weeks)	TBIL, AST, ALT, ALP, LDH and gamma-GT; ALB↑; LPO↓; GSH, SOD and GPx↑; alpha-fetoprotein and caspase-3↓	Anti-oxidative stress; anti-inflammation; anti-apoptosis	Khaled et al. (152)
	Naringin	Grapefruit (*C. paradisi*), *C. aurantium*, and *C. maxima*	Diclofenac-induced liver injury in Wistar rats	20 mg/kg (4 weeks)	Serum ALT, AST, LDH, ALP, GGT, TBIL, TNF-α and IL-17↓; liver LPO↓; liver p53 andcaspase-3 mRNA↓; serum IL-4, liver GSH, GPx and SOD↑; histological, hydropic degeneration, cytoplasmic vacuolization, apoptosis, and focal necrosis, inflammatory cells’ infiltration↓	Anti-oxidative stress; anti-inflammation; anti-apoptosis	Hassan et al. (153)
	Naringin	*Citrus grandis* (L.)	APAP-induced injury in primary hepatocytes and HepG2 cells	1, 10 and 100 mM (24 h pre 8 h)	Cell viability↑; ALT, AST and LDH↓; GSH and SOD↑; phase II enzymes (UGT1A1, UGT1A3, UGT1A6, SULT1A1, SULT2A1, GSTa1 and GSTm1)↓; significant loss of MMP, mitochondrial depolarization, and mitochondrial fission (fusion proteins Mfn1 and Opa1)	Anti-oxidative stress; AMPK/Nrf2 pathway↑	Wu et al. (156)
	Naringin	Grapefruit and other citrus fruits	APAP-induced liver injury in C57BL/6 mice	30, 60 and 120 mg/kg (7 days)	CHAC2 and Nrf2↑; CAT, SOD and GSH↑; proinflammatory cytokines↓; apoptotic pathways↑	Anti-oxidative stress, via CHAC2-mediated activation of the Nrf2 pathway; anti-inflammation	Zhai et al. (157)
	Narirutin	Citrus peels	APAP-induced liver injury in mice	50 mg/kg (24 h)	Serum GPT and GOT1/AST↓; SOD, CAT and GPx↓; CYC/Cyt c↓	Maintain mitochondrial function; anti-oxidative stress; PPP3/calcineurin-TFEB-ALP axis↑	Fang et al. (161)
	Apigenin	Fruits and vegetables	APAP-induced liver injury in mice	20 and 80 mg/kg (7 days)	ALT, AST, MDA and MPO↓; GSH and ROS↑; SIRT1↑; deacetylated p53↑; p65↓	Anti-oxidative stress; anti-inflammation; promote autophagy; NRF2 pathway↑; SIRT1-p53 axis↑	Zhao et al. (163)
	Apigenin	Fruits and vegetables	Acetaminophen-induced liver injury in mice	100 and 200 mg/kg (7 days)	Serum AST and ALT↓; liver necrosis↓; hepatic GR activity↑; GSH↑; MDA↓	Anti-oxidative stress	Yang et al. (164)
	Kaempferol	Tea, tomatoes and grapefruit	INH- and RIF-induced liver injury in SD rat	1.89 and 3.78 mg/kg (2 and 3 weeks)	CYP2E1↓; AST, ALT and GSP↓; GSH↑; MDA↓	Anti-oxidant defense	Shih et al. (166)
	Kaempferol	Tea, tomatoes and grapefruit	Propacetamol-induced liver injury in mice	62.5, 125 and 250 mg/kg (30 h)	ALT, AST and DNA fragmentation↓; TBARS and 3-NT; CYP2E1↓; UGT1A1, SOD, GPx, CAT and GHS↑; Nrf2 and GCLC↑; TNF-α and IL-6; phosphorylations of JNK and ERK↓; Bax/Bcl-2 ratio and caspase 3 activation↓	Anti-oxidative stress; anti-inflammatory; anti-apoptotic activities	Tsai et al. (37)
	Kaempferol	Tea, tomatoes and grapefruit	APAP--induced liver injury in rat	250 g/kg (7 days)	TNF-a, IL-6, caspase-3, ALT, AST and c-GT↓; GSH and SOD↑; MDA and ROS↓; Bcl-2↑; Bax and cleaved Bax↓; CYP2E1 and SIRT1↓; acetylation of all SIRT1 targets including PARP1, p53, NF-jB, FOXO-1 and p53↓	Anti-oxidant stress; anti-inflammation; anti-apoptotic effects.	BinMowyna et al. (168)
	Hesperetin	Citrus fruits	APAP-induced liver injury in mice/AML12 hepatocyte	5, 10 and 30 mg/kg (24 h, 3 times)/1, 3 and 10 μM (24 h)	*In vivo*, ALT, AST, ALP and LDH↓; caspase3↓; GSH, SOD, CAT and HO-1↑; MDA↓; infiltration of macrophages and neutrophils; p38 and p65↑; TNF-α and IL-1β↓. *In vitro*, ROS production↓; MDA↓; GSH↑; HO-1↑	Anti-oxidant stress; anti-inflammation; anti-apoptotic effects.	Wan et al. (170)
	Alpha-mangostin	Mangosteen (*Garcinia mangostana*)	APAP--induced liver injury in mice	100 and 200 mg/kg (24 h)	AST and ALT↓; GSH↑; MDA↓; TNF-α and IL-1β↓; LC3 and BNIP3↓; Bcl-2↑; Bax and cleaved caspase 3 proteins↓; p62↓; p-mTOR, p-AKT and LC3 II /LC3 I ratio↑	Anti-oxidant defense; anti-inflammation; anti-apoptosis; activation of autopsy regulation of Akt/mTOR pathway	Yan et al. (172)
	Alpha-mangostin	Pericarp of mangosteen	APAP--induced liver injury in mice	12.5 and 25 mg/kg (7 days)	ALT and AST↓; GSH and SOD↑; MDA↓; Histologically, inflammation (Kupffer cell activation) and cell necrosis areas↓; IL-6, IL-1β, TNF-α and tissue mRNA expression↓; iNOS mRNA↑; IκB-α↓; phosphorylation of ERK, JNK, and p38↓	Anti-oxidant and anti-inflammatory properties mediated through NF-κB and MAPK signaling pathways.	Fu et al. (53)
	Davallialactone	Mushroom Inonotus xeranticus	APAP-induced liver injury in mice	10 mg/kg (6 h)	GOT and GPT↓; ATP and GSH↑; peroxynitrite and 4-HNE formations↑; GSH/GSSG ratio↑; ROS	Anti-oxidative stress; JNK/ERK signaling pathway↓	Noh et al. (173)
	Saponarin	*Gypsophila trichotoma*	APAP-induced injury in rat hepatocytes	60–0.006 g/mL (1 h)	Cell viability↑; LDH leakage and MDA↓; GSH↑	Anti-oxidant defense	Simeonova et al. (174)
	Saponarin	Gypsophila trichotoma	APAP-induced liver injury in rat	80 mg/kg (7 days)	MDA↓; LPO↓; GSH↑	Anti-oxidant defense	Simeonova et al. (174)
Glycosides	Amygdalin	Bitter Apricot Seed	Apap-induced liver injury in C57BL/6 mice	2.5 and 5 mg/kg (12 h)	ALT/AST↓; tissue necrotic area↓; neutrophils and macrophages; IL-6, TNFa, and IL-1b↓; SOD↑; MDA↓	Anti-oxidant and anti-inflammatory properties; Nrf2/NQO1/HO1 signaling pathway↑; JNK/RIP3/MLKL signaling↓	Zhang et al. (176)
	Ginsenosides	*Panax ginseng*	APAP-induced liver injury in mice	150 and 300 mg/kg (7 days)	AST, ALT and hepatic MDA↓; SOD and GSH↑; 4-HNE↓; CYP2E1↓; TNF-alpha, IL-1, Bax, Bcl-2 and COX-2↓; hepatocyte necrosis and inflammatory cell infiltration↓	antioxidant and anti-inflammatory properties; Nrf2/NQO1/HO1 signaling pathway↑	Yang et al. (134); Xu et al. (178)
	Ginsenosides Rg1/Rh1	Ginseng	APAP-induced liver injury in mice	Rg1 (10, 20 and 30 mg/kg) and Rh1 (10, 20 and 30 mg/kg) (7 days)	GSH and SOD↑; MDA↓; GOT and GPT↓; TNF-α, IL-6, and IL-1β↓; Bax↓ and Bcl-2↑	Anti-oxidant defense; anti-inflammation; anti-opoptosis	Bi et al. (179)
	Ginsenosides Rg3	Ginseng	APAP-induced liver injury in mice	5, 10 and 20 mg/kg (6 h)	ALT, ALP, AST and LDH↓; GHS and GPx↑; MDA↓; cell apoptosis and inflammatory infiltration↓	Anti-oxidant, anti-apoptotic and anti-inflammatory effects	Gao et al. (182)
	Ginsenosides Rg3	Korea red ginseng	APAP-induced liver injury in mice	10 and 20 mg/kg (7 days)	GSH↑; CYP2E1↓; MDA and 4-HNE in a dose-dependent manner↓; LPO and ROS↓; hepatocyte necrosis, apoptosis and inflammatory infiltration of lymphocytes↓; Bax↓ and Bcl-2↑; IKKα, IKKβ and I-κBα↓	Anti-oxidant, anti-apoptotic and anti-inflammatory effects; NF-κB signaling; PI3K/AKT signaling.	Zhou et al. (183)
	Ginsenosides Rg5	Black ginseng	Acetaminophen-Induced Hepatotoxicity in Mice	10 and 20 mg/kg (7 days)	TNF-α, IL-1β, MDA, 4-HNE and CYP2E1↓; COX-2 and iNOS↓; Bcl-2↑; Bax↓; caspase-3/8/9↓	Anti-oxidant, anti-apoptotic and anti-inflammatory effects	Wang et al. (52)
	Ginsenoside Rk1	Ginseng	APAP-induced liver injury in mice	10 and 20 mg/kg (7 days)	ALT, AST, TNF and IL-1↓; SOD and GHS↑; MDA, 4-HNE and CYP2E1↓; Bcl-2↑ and Bax↓; tissue necrosis and inflammatory infiltration↓; 3-nitrotyrosine↓	Anti-oxidation, anti-apoptosis, anti-inflammation, and anti-nitrative effects	Hu et al. (184)
	Ginsenoside Rk3	*Panax notoginseng*	APAP-induced liver injury in mice	25 and 50 mg/kg (7 days)	AST and ALT↓; TNF-α, IL-6 and IL-1β↓; SOD and GHS↑; MDA↓; CYP2E1↓; Nrf2 and HO-1↑; LC3 II/LC3 I, Beclin 1, ATG 5, ATG 7 and ATG 12↑; P62↓	Anti-oxidant; anti-inflammation; activate autophagy.	Qu et al. (58)
	Ginsenoside Rb1	*Panax ginseng*	APAP-induced liver injury in mice	10 mg and 20 mg/kg (1 weeks)	ALT and AST↓; GSH↑; TNF-α, IL-1β, iNOS and COX-2↓; phosphorylation of MAPK and PI3K/Akt↓; NF-κB↓	Inhibit inflammatory response mediated by the MAPK and PI3K/Akt signaling pathways; antioxidant defense.	Ren et al. (185)
	Compound K	Fermented ginseng	APAP-induced liver injury in rat	50 mg/kg (8 days)	ALT and AST↓; genes related to JNK, GST↓; phosphorylation of JNK↓	Anti-inflammatory by inhibiting JNK signaling.	Igami et al. (186)
	Jujuboside B	Ziziphi Spinosae Semen	APAP-induced liver injury in C57BL/6 J mice	20 and 40 mg/kg (7 days)	CYP2E1↓; pro-inflammatory cytokines↓; Nrf2 nuclear translocation of Nrf2↑; HO-1 and NQO-1↑	Anti-oxidant defense and anti-inflammation; regulation of the Nrf2-STING pathway	Wang et al. (187)
Terpenes	Taraxasterol	*Taraxacum officinale*	APAP-stimulated liver damage in Kunming mice/AML12 cells	2.5, 5 and 10 mg/kg (7 days)/5, 10 and 20 μg/mL (12 h)	Serum AST and ALT↓; SOD, CAT and GSH↑; ROS↓; Nrf2 and HO-1↑; JNK phosphorylation↓; Bax/Bcl-2 ratio and caspase-3↓	Anti-oxidative stress; anti-inflammatory response; anti-apoptosis; Nrf2/HO-1 pathway↑	Ge et al. (189)
	Kahweol	Coffee	APAP-induce liver damage in C57BL/6 N mice	20 mg/kg (36 h)	LPO↓; GSH↑; Nrf2↑; NF-κB; infiltration of neutrophils and macrophages↓	Inhibit oxidative stress, ER stress, and inflammation.	Kim et al. (191)
	γ-oryzanol (ORY)	Rice bran	APAP-indued liver livery in mice/HL-7702 cells	7, 14 mg/kg (7 days); 5 and 10 μg/ mL for 24 h	Nrf2↑; HO-1, NQO1, GCLC and GCLM↑; modulated the AMPK/GSK3β axis; NF-κB p65 subunit↓; iNOS and COX-2↓; TNF-α, IL-1β, IL-6 and NO↓	Anti-oxidant defense and anti-inflammation; modulation of AMPK/GSK3β/Nrf2 and NF-κB signaling pathways	Shu et al. (151); Gomes et al. (194)
Carotenoids	Astaxanthin	Crustaceans and fish	APAP-induced liver injury in C57BL/6 mice/L02 liver cells	10 mg/kg (14 days)/ (25 and 50 μM) for 24 h	ALT, AST and LDH↓; IL-1β and IL-6↓; MDA and LPO↓; Nrf2, HO-1,SOD and PHx4↑; LC3B/LC3A ratio↑; p62↓; SLC7A11, GPX4, FTH1 and FTL1↑	Anti-oxidation; anti-apoptosis; anti-inflammation; inhibition of ferroptosis; activation of autophagy; maintain mitochondrial function, NF-κB pathway↓; Nrf2/HO-1 antioxidant pathway↑	Cai et al. (64)
	Astaxanthin	Crustaceans and fish	APAP-induced liver injury in C57BL/6 mice	30 and 60 mg/kg (14 days)	ALT, AST, hepatic necrosis, ROS generation and LPO↓; GSH and SOD↑; phosphorylation of JNK, ERK and P38↓; TNF-α and TRAF2↓	Anti-oxidation; anti-apoptosis; JNK signaling pathway↓	Zhang et al. (197)
	Astaxanthin	Crustaceans and fish	DOX-induced liver injury in mice	50 and 100 mg/kg (21 days)	ALT, GOT, ALP and TBIL↓; MDA and ROS↓; SOD, CAT and GPX↑; Keap1↓; Nrf2↑; ERK↑	Anti-oxidant defense via modulating Keap1/Nrf2 signaling pathway	Ma et al. (198)
	Lycopene	Tomatoes	APAP-induced liver injury in C57BL/6 mice	10 and 100 mg/kg (1 h)	GSSG↓; tGSH and CAT↑; protein carbonylation↓; MMP-2↓	Anti-oxidant defense	Bandeira et al. (200)
	Torularhodin	Red wine	APAP-induced liver injury in C57BL/6 mice	9, 13. 5 and 18 mg/kg (10 days)	SOD, GSH-Px↑; MDA; TNF-α, IL-1β and IL-6↓	Anti-oxidation; anti-inflammation; regulation of PI3K/Akt/mTOR and Nrf2/HO-1 signaling pathway	Li et al. (202)

#### 4.2.1 Phenols

##### 4.2.1.1 Resveratrol (RESV)

Resveratrol (RESV) is a natural nonflavonoid polyphenol that is present in the fruits of many plants, such as grapes and peanuts. In a cat model of As_2_O_3_-induced liver injury, RESV treatment increased antioxidant enzyme activity and decreased As_2_O_3_-induced ROS and malondialdehyde (MDA) production. Additionally, RESV alleviated the decrease in the reduced GSH to oxidized GSH ratio and the arsenic retention in liver tissue caused by As_2_O_3_ (116). These findings elucidated that the protective effects of RESV on As_2_O_3_-induced oxidative stress and hepatotoxicity were realized through decreasing the retention of arsenic and improving the redox status of liver tissue (116). In the APAP-induced C56BL/6 mouse liver injury model, long-term RESV treatment improved liver injury caused by APAP poisoning, restored tissue characteristics, ultrastructure and serum biochemical indicators [albumin and alanine aminotransferase (ALT)], and restored liver cell recovery indicators, such as Ck18- and F4/80-positive cells (117).

##### 4.2.1.2 Gallic acid (GA)

Gallic acid (GA), a small phenolic acid, is a hepatoprotective active component of several food extracts, such as onion, date, berries, grape, apple, tea leaves and pomegranate. Increasing evidence supports the antioxidant and anti-inflammatory properties of GA through its influence on cytoprotective pathways. Experimental studies have shown that GA is beneficial for the treatment of liver damage caused by acetaminophen and antituberculosis drugs (isoniazid and rifampicin) (118, 119). GA treatment can significantly reverse the increase in liver enzyme markers and inflammatory mediators TNF-*α* and LPO induced by paracetamol and improve the measured antioxidant status of paracetamol-stimulated mice, suggesting that GA treatment has potential antioxidant and anti-inflammatory effects (118). In a Wistar rat model of hepatotoxicity induced by isoniazid and rifampicin, GA effectively prevents the hepatotoxicity induced by isoniazid and rifampicin, improves oxidative stress balance by activating Nrf2, which led to increased level of GSH, PRDX6, GPx, SOD and CAT, and inhibits the NF-κB signaling pathway, downregulating the level of TLR, NOS2, IL-1β, IFN-ɣ, and high mobility group box-1 protein (HMGB)-1 *in vivo* (119).

##### 4.2.1.3 Capsaicin (CAP)

Capsaicin (CAP) is an ingredient of chili peppers and has complex pharmacologic effects (120). Antunes et al. found that oral supplementation with CAP attenuated oxidative stress and inflammation in a murine model of food allergy (121). Zhang et al. reported that CAP has antioxidative and anti-inflammatory effects on concanavalin A-induced hepatic injury in mice (122). Zhan et al. established an APAP-induced model of ALI in mice and observed the protective effect of CAP on APAP-induced ALI (123). Research showed that CAP pretreatment significantly attenuated ALI and improved oxidative stress-associated indicators; CAP pretreatment downregulated the expression of proinflammatory cytokines through the HMGB1/TLR4/NF-κB signaling pathway; in addition, CAP pretreatment alleviated hepatocyte apoptosis by inhibiting the expression of B-cell lymphoma-2 (Bcl-2)-associated X, caspase-3 and cleaved caspase-3 (123).

##### 4.2.1.4 Maltol (MAL)

Maltol (MAL) is a flavor enhancer, a natural antioxidant, and one of the Maillard reaction products of heated-processed ginseng (124). MAL was also found in roasted Korean ginseng roots (125). Wang et al. reported that MAL inhibited oxidative stress and pyroptosis and further reduced cisplatin-induced apoptosis. The results of this study indicated that MAL protects against cisplatin-induced intestinal toxicity by reducing the release of ROS and inhibiting the activation of apoptosis (126). In addition, Wang et al. investigated the protective effect and elucidated the mechanisms of action of MAL on APAP-induced liver injury *in vivo*. These findings suggested that MAL has a significant liver-protective effect, which may be related to antioxidant defense, anti-inflammatory effects, and antiapoptotic effects, which may be achieved through the regulation of the PI3K/Akt signaling pathway (127).

##### 4.2.1.5 6′-O-Caffeoylarbutin (CA)

6′-O-Caffeoylarbutin (CA) is an arbutin derivative and is the most abundant compound in Que Zui tea (QT). The protective effect of CA against acute liver damage induced by APAP was investigated *in vivo* and *in vitro*. The results showed that CA pretreatment could significantly reduce the level of liver functional enzymes in HepG2 cells and mice induced by APAP, significantly improve the measured antioxidant status, and increase the amount of Nrf2 protein in the nucleus, improve ARE- dependent anti-oxidant protein expression and CA also alleviated the oxidative stress induced by APAP by activating the Nrf2 signaling pathway. Furthermore, CA pretreatment significantly reduced the release of proinflammatory cytokines induced by APAP, indicating that CA mitigated liver damage by inhibiting the inflammatory response (128).

##### 4.2.1.6 Caffeic acid (CAF)

Caffeic acid (CAF), a polyphenolic compound, is commonly present in various edible plants, such as fruits, coffee, and honey. CAFs have been shown to reduce liver toxicity in rats by restoring liver enzymes (129). CAFs have also been shown to reduce APAP-induced liver damage by restoring GSH and liver enzymes while also reducing myeloperoxidase (MPO) activity, ROS levels, and histopathological damage. In addition, CAFs activate Nrf2 in liver cells by blocking Keap1 binding to Nrf2 (130). Moreover, CAFs help detoxify APAP-induced liver damage by inhibiting ERK1/2-mediated Egr1 transcriptional activation (131).

##### 4.2.1.7 Other phenolic compounds

Other phenolic compounds of food origin, such as cajaninstilbene acid (CSA), a major stilbene compound extracted from pigeon bean [*Cajanus cajan* (L.) Millsp.] leaves. Without affecting APAP metabolic activation, CSA blocks the ongoing JNK-Sab ROS activating ring and alleviates oxidative stress (132). CSA was revealed to promote mitochondrial quality control, including mitochondrial biogenesis and mitophagy. Further mechanistic investigations showed that CSA alleviates APAP-induced oxidative stress and enhances mitochondrial quality control through sestrin2/AMPK activation, thereby protecting against AILI (132).

Chlorogenic acid (CGA), a polyphenolic compound, is abundant in coffee, apples, blueberries, tea, and honeysuckle (133); genistein, from soybean (134); daidzein, from soybean (135); polyphenols, from tofu (136); guavinoside B, from *Psidium guajava* (137); formononetin, from legume (138); heilaohusuin B, from *Kadsura coccinea* (139); and chebulinic acid, from *Terminalia chebula* fruit (140) were investigated and confirmed to inhibit oxidative stress, providing protective effects against drug-induced liver toxicity. In addition, guavinoside B (137) and formononetin (138) were found to exhibit anti-inflammatory and antiapoptotic effects of formononetin (138). Further mechanistic studies indicated that GAA (133), guavinoside B (137), heilaohusuin B (139), chebulinic acid (140), had potent hepatoprotective effects through the regulation of the Nrf2 signaling pathways. Furthermore, guavinoside B exerts protective effects via Nrf2 and JNK signaling pathways, which led to a decrease in intracellular ROS levels; the elevated levels of ALT, AST, ROS, MDA, and TNF-*α* induced by APAP were reduced, while the decreased levels of SOD and GSH were restored; the expression levels of Nrf2, GCLC, and NQO1 were upregulated, and the gene expression of p-JNK was downregulated (137); CGA has a significant protective effect against APAP hepatotoxicity via many mechanisms, including blocking the expression of CYP2E1 and CYP1A2 enzymes, alleviating mitochondrial damage in the liver, reducing mitochondrial heat shock protein 60 (HSP60) production, and inhibiting the MAPK, TLR3/4 and NF-κB pathways (133). Long-term intake of CGA from food sources can trigger PINK1-dependent mitophagy (mitochondrial autophagy), thereby suppressing liver cell death to reduce APAP hepatotoxicity (31).

#### 4.2.2 Flavonoids

##### 4.2.2.1 Cynarin (Cyn)

Cynarin (Cyn), derived from hydroxycinnamic acid, is present in the food-derived Illyrian thistle (*Onopordum illyricum* L.), and it displays significant antioxidant, anticholinergic, radical-scavenging, and metal-binding properties due to its bioactive functional groups (141). Cynarin promotes Nrf2 dissociation from Keap1, enhances Nrf2 nuclear translocation and downstream antioxidant protein transcription, thereby inhibiting lipid peroxidation. In addition, Cyn activates the adenosine monophosphate-activated protein kinase (AMPK)/sirtuin (SIRT)3 signaling pathway with a protective effect on APAP-induced acute lung injury (ALI). These findings suggest that Cyn enhances Keap1/Nrf2-mediated defense against lipid peroxidation by activating the AMPK/SIRT3 signaling pathway, thereby alleviating APAP-induced ALI (142).

##### 4.2.2.2 Mangiferin (MAN)

Treatment with mangiferin (MAN), present in mangos, restored the GSH depletion caused by APAP overdose, reducing the formation of APAP-Cys adducts and promoting protection. MAN treatment downregulated p-JNK and activated AMPK and the expression of inflammation-related genes, which suggested that MAN plays a protective and therapeutic role in APAP-induced hepatotoxicity by improving APAP metabolism and APAP-Cys adduct formation, followed by JNK-mediated oxidative stress and inflammation (143).

##### 4.2.2.3 Epicatechin (Epi)

Epicatechin (Epi) is commonly present in grapes, cola nuts, straw berries and red wine. Epi has been reported to improve streptozotocin- (STZ) (144), methotrexate- (MTX) (145), APAP- (146) and doxorubicin (DOX) (147)-induced liver injury. In an STZ-induced diabetic rat model, (−)-epicatechin treatment decreased the concentrations of thiobarbituric acid reactive substances (TBARS) and hydroperoxides (HPs) and improved the antioxidant status of diabetic tissues such as the liver, kidney and heart (144). In a rat model of MTX-induced diabetes, Epi pretreatment reduced liver dysfunction by improving the antioxidant defense system and anti-inflammatory effects and alleviating histopathological damage in the context of MTX hepatotoxicity (145). In addition, Wu et al. indicated that Epi inhibits acute liver injury induced by APAP by suppressing inflammatory factors to alleviate the immune response and pathological damage and downregulating the mitochondrial apoptosis pathway to alleviate liver injury (146). Similarly, Epi may be an effective chemoprophylencer against DOX-induced hepatotoxicity by enhancing the antioxidant defense system and reducing the effects of inflammation and apoptosis (147).

##### 4.2.2.4 Naringin (Nar)

Naringin (Nar) is a dihydroflavonoid extracted primarily from *Citrus grandis* (L.) Osbeck and the immature or almost mature dried outer peel of grapefruit (*C. paradisi* Macfad). Elsawy et al. reported that Nar can significantly reduce the upregulation of MTX-induced liver injury markers in rats, reduce oxidative stress, and protect liver cells from MTX-induced damage (148). Nar also significantly decreased serum toxicity markers, increased antioxidant enzyme activities, and regulated inflammation, apoptosis, autophagy, and oxidative DNA damage induced by cyclophosphamide (149, 150). In experimental studies of Nar counteracting doxorubicin- (151), Taxol- (152), and diclofenac (153)-induced liver injury *in vivo*, it was also confirmed that Nar exerts hepatoprotective effects through functions such as antioxidation, anti-inflammation, and apoptosis inhibition. In addition, Nar can ameliorate APAP-induced oxidative stress and liver tissue damage *in vivo* (154, 155). Moreover, several *in vitro* and *in vivo* studies have extensively investigated the mechanisms by which Nar alleviates APAP-induced liver injury. Wu et al. found that APAP reduced the concentrations of GSH and SOD in liver cells while increasing MDA and ROS levels, as well as the expression of CYP2E1. However, Nar pre-treatment reversed these indicators, suggesting that Nar has a protective effect against APAP-induced liver cell damage. Further mechanistic studies revealed that Nrf2 plays a crucial role in regulating the expression of various antioxidant enzymes. Nar pre-treatment induced an upregulation of Nrf2 protein levels and phosphorylation of AMPK. Pre-treatment with dorsomorphin (an AMPK inhibitor) effectively blocked Nar-mediated Nrf2 activation and AMPK phosphorylation, while brusatol (an Nrf2 inhibitor) had no significant effect on Nar-mediated AMPK phosphorylation, indicating that AMPK can act as an upstream regulator of Nrf2. These experimental results suggest that Nar alleviates APAP-induced hepatocyte and mitochondrial injury by activating the AMPK/Nrf2 pathway to reduce oxidative stress *in vitro* (156). Zhai et al. indicated that Nar may be a potent activator of cation transport regulator-like 2 (CHAC2), alleviating APAP-induced hepatitis through CHAC2-mediated Nrf2 pathway activation and inhibiting hepatic oxidative stress, inflammation, and hepatocyte apoptosis (157). Interestingly, a previous report highlighted the therapeutic potential of autophagy in the treatment of APAP-induced liver injury (158, 159). Transcription factor EB (TFEB) regulates a set of genes involved in autophagy and lysosome biogenesis, and dephosphorylation of TFEB by PP3P/calcineurin phosphatase hydrolysis mediates its transcriptional activity (160). Thus, triggering TFEB-mediated macroautophagy/autophagy-lysosomal pathway (ALP) activation potentially provides a therapeutic option for APAP overdose. Based on the above mechanism, Fang et al. reported that Nar protects against APAP-induced liver injury by activating the PPP3/calcineurin-TFEB-ALP axis, indicating that Nar may be a potential agent for treating APAP overdose (161). Interestingly, The mixture of naringin and naringenin seemed to be more effective in enhancing organ function and maintaining structural integrity. In summary, naringin and naringenin are recommended for their hepatoprotective benefits by strengthening the body’s antioxidant defense system, lowering inflammation, and inhibiting apoptosis (151). In addition, the administration of a combination of hesperidin and naringin was found to be the most powerful in potentially counteracting liver injury and toxicity induced by diclofenac through enhancing the antioxidant defense system, anti-inflammatory properties, as well as suppressing oxidative stress and apoptosis (152).

Currently, no significant adverse reactions have been reported with Nar, and various studies have confirmed the benefits of consuming water infused with grapefruit peel in everyday life. In summary, all of this suggests that Nar could be an effective element in treating DILI.

##### 4.2.2.5 Apigenin (API)

Apigenin (API) is one of the most abundant flavones in parsley, onions, oranges, tea, and chamomile (162). *In vivo* studies have shown that API has beneficial effects, such as antioxidation, anti-inflammation, anti-apoptosis and autophagy-promoting effects (163, 164). Further research on the mechanism by which API alleviates APAP-induced liver injury revealed that API alleviates APAP-induced liver injury by regulating the SIRT1-p53 axis, thereby promoting APAP-induced autophagy and improving the APAP-induced inflammatory response and oxidative stress damage (163). Overall, API has a potential protective effect in drug-induced liver injury (DILI). However, celery is a high-fiber vegetable that may increase the burden on the liver. Despite celery being rich in API, it is noteworthy that it is not recommended for patients with cirrhosis.

##### 4.2.2.6 Kaempferol (Kae)

Kaempferol (Kae) is one of the most common aglycone flavonoids in the form of glycosides. It is found in a wide variety of plant foods and plant-based supplements, including kale, beans, tea, spinach, and broccoli (165). In 2013, Shih et al. first reported that Kae, as an adjuvant, prevents the CYP2E1-mediated hepatotoxicity induced by anti-tuberculosis drugs (166). Mechanistically, Kae enhances antioxidant defense against isoniazid/rifampin (INH/RIF)-induced hepatotoxicity (167). Interestingly, Tsai et al. indicated that Kae protects the liver against propacetamol-induced damage not only through antioxidative and anti-inflammatory effects but also through antiapoptotic effects (167). BinMowyna et al. highlighted a novel mechanism by which Kae decreases the acetylation of all SIRT1 targets to mediate antioxidant, anti-inflammatory and antiapoptotic effects (168).

##### 4.2.2.7 Hesperetin (Hst)

Hesperetin (Hst) is a dihydrogen flavonoid extracted from the citrus fruits of Rutaceae plants and the glycosyl ligand of hesperidin (169). Recently, Wan et al. showed that Hst pretreatment alleviated AILI both *in vivo* and *in vitro*. The hepatoprotective effect of Hst is achieved by alleviating oxidative stress, inhibiting the inflammatory response and inhibiting apoptosis, and its anti-inflammatory effect could be linked to the inhibition of TLR4 signaling pathway activation (170).

##### 4.2.2.8 α-Mangostin (α-MA)

α-Mangostin (α-MA), a flavonoid, is one of the significant phytochemical components found in the tropical fruit mangosteen (*Garcinia mangostana*) and in mangosteen (*G. mangostana*) (171). Fu et al. revealed that the hepatoprotective effect of α-MA may be mediated by inhibiting APAP-mediated MAPK activation (172). Another study by Yan et al. showed that α-M exhibits significant hepatoprotective effects through its antioxidant and anti-inflammatory properties *in vivo* and confirmed that the detoxification effect of α-MA on APAP-induced ALI is related to the regulation of the Akt/mTOR pathway (52).

##### 4.2.2.9 Others

In addition, davallialactone (DAVA), isolated from *I. xeranticus*, had protective effects against APAP overdose-induced liver injury via its antioxidant activity (173). Saponarin (Sap), which is isolated from *Gypsophila trichotoma*, also exerts antioxidant and hepatoprotective effects on acetaminophen-induced liver injury both *in vitro* and *in vivo* (174).

#### 4.2.3 Glycosides

##### 4.2.3.1 Amygdalin (AMG)

Amygdalin (AMG) is a natural compound isolated from bitter almond seeds that has broad anti-inflammatory and analgesic activities. AMG can alleviate CCL4- and APAP-induced liver damage (175, 176). AMG was found to have a protective effect on APAP-induced liver injury through its anti-inflammatory and antioxidant effects (176). Further mechanism studies revealed that amygdalin reduced the expression of Nrf2 and its downstream proteins NQO1 and HO1. Nrf2 is recognized as the primary transcription factor for maintaining cellular redox homeostasis and combating oxidative stress. The downregulation of Nrf2 by amygdalin is beneficial for alleviating acute liver injury induced by acetaminophen (APAP). Additionally, amygdalin treatment significantly increased the phosphorylation levels of AKT and JNK. The activation of p-JNK can enhance the nuclear translocation of Nrf2 and increase its expression. As an upstream factor of JNK, the activation of p-AKT also promotes Nrf2’s nuclear expression. This suggests that amygdalin may resist oxidative stress and mitigate APAP-induced liver injury through the AKT/JNK/Nrf2 pathway. Moreover, amygdalin treatment reduced indicators related to cell death, such as terminal dUTP nick end labeling (TUNEL), and markers associated with necroptosis, including p-MLKL (Mixed Lineage Kinase Domain-Like) and RIP3 (Receptor-interacting Protein Kinase 3). In summary, these results indicate that amygdalin exerts a protective effect against APAP-induced liver injury through its anti-inflammatory and antioxidant properties, linked to increased AKT phosphorylation and the inhibition of the JNK/RIP3/MLKL signaling pathway (176).

##### 4.2.3.2 Ginsenosides

Ginsenosides are a class of bioactive substances extracted from the roots of the medicinal plant ginseng, which has been used in traditional Chinese medicine for centuries. The medicinal properties of ginseng have been extensively documented in relation to the central nervous, cardiovascular, endocrine, and immune systems. Additionally, ginseng possesses anti-cancer, anti-stress, antioxidant, and antiviral properties. The positive effects of ginseng are attributed to its diverse pharmacologically active components, with most of the pharmacological benefits linked to ginsenosides. Extensive research has explored the hepatoprotective properties of ginsenosides, addressing mild to severe liver damage and liver fibrosis caused by various etiologies. Previous studies have shown that various components, including ginsenosides Rg1, Rg3, Rg5, Rk1, Rk3, and Rb1 and compound K, significantly alleviate liver damage in both *in vivo* and *in vitro* models of DILI through different mechanisms of action (177–186). Ginsenosides can improve the hepatotoxicity and antioxidant activity of APAP and inhibit the inflammatory response induced by APAP (177–179, 182–186). Furthermore, ginsenosides inhibited the activation of apoptotic signaling pathways by increasing Bcl-2 expression and decreasing the protein levels of Bax and caspase-3 (186). Bi et al. suggested that ginsenoside Rg1 and ginsenoside Rh1 exert protective effects against APAP-induced liver damage through their antioxidative, antiapoptotic, and anti-inflammatory activities (179). Interestingly, Gao et al. reported that ginsenoside Rg1 activates the p62-Keap1-Nrf2 signaling pathway to exert antioxidant effects, thereby protecting against cisplatin-induced liver injury (180). Ginsenoside Rg3 is a saponin isolated from *Panax ginseng* C. A. Meyer, *Panax notoginseng*, or *Panax quinquefolius* L. (181) Ginsenoside Rg3 treatment significantly relieved APAP-induced hepatic tissue inflammation and oxidative stress. Moreover, molecular docking studies have shown that ginsenoside Rg3 can bind to NLRP3, indicating its anti-inflammatory effects (182). Zhou et al., revealed that 20(*R*)-Rg3 played an important role in alleviating APAP-induced liver injury by inhibiting oxidative stress, improving inflammatory response, alleviating apoptosis and necrosis, and regulating PI3K/AKT pathways-mediated Bax/Bcl-2 and NF-κB signal cascade. Hu et al. reported that the hepatoprotection of the ginsenoside Rk1 may be due to its antioxidative, antiapoptotic, anti-inflammatory, and antinitration effects (184). Additionally, Ren et al. suggested that Rb1 has a significant hepatoprotective effect on APAP-induced ALI, partly through modulation of the inflammatory response mediated by the MAPK and PI3K/Akt signaling pathways (185). In addition, Igami et al. evaluated the effect of fermented ginseng (FG) containing a high concentration of complex K on APAP-induced liver and HepG2 cell damage in rats. FG rich in complex K reduced serum AST and ALT levels in rats. DNA microarray analysis suggested that complex K in FG may play an important role in APAP-induced liver injury by inhibiting JNK signaling pathways in the liver (186). In addition, the ability of ginsenoside Rg5 to exert hepatoprotection by mainly inducing an antiapoptotic effect mediated through caspases was investigated (52). Hepatoprotection by the ginsenoside Rk3 in APAP-induced hepatic toxicity was mainly dependent on its antioxidative and anti-inflammatory effects and continuous activation of autophagy (58).

##### 4.2.3.3 Jujuboside B (JuB)

Jujuboside B (JuB) is the main saponin in jujube kernels. Wang et al. reported that JuB pretreatment reversed the decrease in CYP2E1 levels, inhibited oxidative stress, reduced the production of proinflammatory cytokines, and alleviated hepatocyte apoptosis. Further mechanistic studies revealed that JuB treatment upregulated total Nrf2 levels, promoted its nuclear translocation, increased the expression of HO-1 and NQO-1, and inhibited the activation of the STING pathway induced by APAP. Moreover, the beneficial effects of JuB were weakened in the presence of DMXAA (a specific STING inhibitor) and ML385 (a specific Nrf2 inhibitor), suggesting that JuB prevents APAP-induced hepatotoxicity through the Nrf2-STING pathway (187).

#### 4.2.4 Terpenes

##### 4.2.4.1 Taraxasterol (TAX)

Taraxasterol (TAX) is a five-ring triterpenoid compound extracted from the edible plant *Taraxacum officinale* (188). Ge and colleagues obtained 24 common targets of taraxasterol and drug-induced liver injury (DILI) from an online database and selected 9 core targets for subsequent enrichment analysis. The results of GO and KEGG enrichment analysis indicated that the core targets play significant roles in oxidative stress, inflammatory response, and apoptosis. *In vivo* experiments confirmed that taraxasterol significantly reduced serum ALT and AST activities induced by APAP, and tissue pathology further verified that taraxasterol alleviated APAP-induced liver injury in mice. Additionally, both *in vitro* and *in vivo* results showed that taraxasterol enhanced antioxidant capacity by increasing GSH and SOD activities while inhibiting the production of ROS and MDA. Further mechanistic studies confirmed that taraxasterol alleviated the oxidative stress response induced by APAP by inhibiting JNK phosphorylation and activating the Nrf2/HO-1 signaling pathway, leading to increased GSH and SOD activities and decreased ROS and MDA levels. The experiments also validated that taraxasterol inhibited the secretion and expression of cytokines IL-1β, IL-6, and TNF induced by APAP, reduced apoptosis in APAP-treated AML12 cells and mouse hepatocytes, significantly lowered the Bax/Bcl-2 ratio, and downregulated the expression of caspase-3. Moreover, taraxasterol exerted a hepatoprotective effect by inhibiting JNK phosphorylation and activating the Nrf2/HO-1 signaling pathway, which was confirmed through network pharmacology analysis. These findings suggest that taraxasterol improves oxidative stress, inflammation, and apoptosis induced by APAP, helping to prevent the progression of drug-induced liver injury (189).

However, this study conducted by Lin and others mainly focused on the Nrf2 protein, emphasizing specifically how the Nrf2 protein mediates the role of TAX in countering APAP-induced liver injury. Research data shows that Nrf2 mediates TAX’s protection against APAP-induced liver injury, and significant attenuation of protective effect of TAXwas observed through knockout of Nrf2 using AAV-Nrf2-KO. Furthermore, depletion of Nrf2 weakened TAX inhibitory effects on APAP-induced oxidative stress and liver inflammation. In addition, inhibition of Nrf2 by ML-383 may also weaken the protective effects of TAX against APAP-induced cell damage, oxidative stress, and secretion of inflammatory factors, suggesting that Nrf2 is involved in regulating the modulation effect of TAX on APAP-induced liver injury. In conclusion, TAX plays a protective role against APAP-induced liver injury by inhibiting oxidative stress and liver inflammation, with an important involvement of Nrf2 in mediating the antioxidant and anti-inflammatory stress effects exerted by TAX (190).

##### 4.2.4.2 Kahweol (KW)

Kahweol (KW), derived from coffee, exhibits antioxidant and anti-inflammatory effects in acute and chronic inflammatory diseases (191). Studies by Kim et al. have indicated that KW has a protective effect against APAP-induced liver toxicity by activating the antioxidant system, inhibiting ER stress-induced liver cell death, and alleviating inflammation mediated by NF-κB (192).

##### 4.2.4.3 γ-Oryzanol (ORY)

γ-Oryzanol (ORY) is a mixture of ferulic acid esters with phytosterols isolated from rice bran oil (193). ORY has been shown to reduce liver damage caused by APAP (194, 195). ORY can reduce APAP-induced hepatocyte apoptosis and subsequent liver injury by regulating the AMPK/GSK3β/Nrf2 and NF-κB signaling pathways (194).

#### 4.2.5 Carotenoids

Previous studies have shown that several carotenoids, such as astaxanthin (ASX), lycopene (LYC), and torularhodin, can alleviate drug-induced liver damage.

##### 4.2.5.1 Astaxanthin (ASX)

ASX is present in aquatic animals, microalgae, flamingoes and Pfaffia (yeast) and possesses a potent antioxidant ability (196). ASX plays a protective role in APAP-induced liver injury by alleviating liver cell necrosis, preventing ROS production, inhibiting oxidative stress and reducing apoptosis. This effect is achieved by blocking the TNF-*α*-mediated JNK signaling pathway and phosphorylating ERK and P38, which have been shown to be effective in preventing and treating liver damage in mice (197). ASX can protect against APAP-induced liver injury by activating the Nrf2/HO-1 pathway, which mainly affects oxidative stress, autophagy, and ferroptosis processes (64). ASX was found to protect the liver against chemotherapeutic drug (doxorubicin)-induced liver injury through the Keap1/Nrf2/HO-1 pathway in mice (198).

It is already recognized that the transcription factor Nrf2 is a key regulator in maintaining cellular redox homeostasis and plays an important role in mediating iron/heme metabolism. The activation of Nrf2 reduces intracellular iron stores, thereby restoring iron homeostasis and limiting the production of reactive oxygen species (ROS). Targeting Nrf2 or its downstream targets as a strategy for disease intervention through the regulation of ferroptosis is promising. There is concrete evidence suggesting that certain dietary compounds can activate Nrf2 to promote ferroptosis, thereby inhibiting APAP-induced liver injury, for example, Li et al. found that Kaempferol reduced liver damage caused by APAP by inhibiting hepatocyte ferroptosis through the activation of Nrf2. Other researchers demonstrated that astaxanthin could relieve APAP-induced liver injury by activating the Nrf2/HO-1 pathway, which inhibits ferroptosis (64, 167) and enhance autophagy (197). To address this, using different types of dietary compounds to jointly activate Nrf2 and inhibit ferroptosis may have a significant synergistic effect in suppressing APAP-induced liver damage. However, the potential side effects of the combined use should also be taken into consideration and need further validation.

##### 4.2.5.2 Lycopene (LYC)

LYC is an exogenous antioxidant that belongs to the carotenoid family and is responsible for the red pigment found in many fruits and vegetables (199). LYC inhibits NADPH oxidase via the protein kinase C (PKC) pathway, reducing ROS production in SK-Hep-1 cells. *In vivo*, LYC reduces oxidative damage by decreasing protein carbonylation, promotes the downregulation of matrix metalloproteinase (MMP)-2, and reduces necrotic areas, thereby ameliorating APAP-induced liver toxicity (200).

##### 4.2.5.3 Torularhodin

Torularhodin, a compound akin to *β*-carotene found in sporidiobolus pararoseus, has notable antioxidant effects by effectively scavenging peroxyl free radicals (201). Torularhodin can inhibit hepatocyte apoptosis, enhance antioxidant enzyme activity, and intervene in DILI by modulating signaling pathways such as the PI3K/Akt/mTOR and Nrf2/HO-1 pathways, suggesting its potential as a preventive strategy for DILI (202).

#### 4.2.6 Combination of diet-derived compounds against DILI

The combined application of different compounds offers stronger protective effects against liver damage compared to the efficacy of single compounds, as shown in Table 3.

**Table 3:** Combination of diet-derived bioactive compounds for the prevention and treatment of drug-induced liver injury.

Combination of compounds	Model	Does (time)	Changes of biological markers	Molecular mechanisms	Authors (Ref.)
Resveratrol (RES) and Quercetin (QUR)	APAP-induced liver injury in rats	RES (30 mg/kg) and QUR (50 mg/kg) for 7 days	TNF-α and IL-6↓; MDA↓; SOD and GPx↑; ALT↓	Anti-oxidative stress; anti-inflammation	AL Humayed et al. (203)
	APAP-induced kidney injury in rats	RES (30 mg/kg) and QUR (50 mg/kg) for 7 days	Urea and creatinine↓; MDA↓; IL-6 and TNF-α↓	Anti-oxidative stress; anti-inflammation; anti-apoptosis	Dallak et al. (204)
Resveratrol (RES) and Luteolin (LUT)	ANIT-induced cholestasis in rats	RES (200 mg/ kg), LUT (200 mg/ kg) and RES + LUT (200 mg/kg + 200 mg /kg) for 7 days	AST, ALT, TBIL, DBIL, γ-GT and ALP↓; SOD and GSH↑; MDA↓; Serum neutrophil infiltration and ANIT-induced necrosis↓	Anti-oxidative stress	Wu et al. (205)
Astaxanthin (Asx) and Capsaicin (Cap)	CCl4-induced liver injury model in rats	Asx (0.5 μmol/kg) and Cap (1 μmol/kg)	AST and ALT↓	Anti-oxidative stress	Fukuta et al. (206)
Navel orange peel extract, Naringin and Naringenin	APAP-induced liver injury model in rats	Naringin (20 mg/kg), Naringenin (20 mg/kg) and Navel orange peel extract (50 mg/kg), every other day for 4 weeks	Liver function indictor: serum AST, ALT, ALP, LDH, GGT and TBIL↓. Oxidative stress indictors: LPO↓; GSH, GST, GPx and SOD↑. Apoptosis indictors: P53, Bax and Caspase-3↓; Bcl↑. Inflammation indictors: TNF-α↓; IL-4↑.	Anti-oxidative stress; anti-inflammation; anti-apoptosis	Ahmed et al. (155)
Naringin and Naringenin	taxol-induced liver injury in rats	Naringin (10 mg/kg), Naringenin (10 mg/kg) and Naringin + Naringenin (10 mg/kg + 10 mg/kg), every other day for 6 weeks	TBIL, AST, ALT, ALO, LDH, and ɣ-GT↓; hepatic LPO↓; liver GHS↑; SOD and GPx↑; alpha-fetoprotein and caspase-3↓	Antioxidant defense; Anti- inflammation; inhibition of apoptosis	Khaled et al. (151)
Naringin and Hesperidin	diclofenac-induced liver injury in rats	Naringin (20 mg/kg), Hesperidin (20 mg/kg) and Naringin + Hesperidin (20 mg/kg + 20 mg/kg), for 4 weeks	ALT, AST, LDH, ALP, GGT and TBIL↓; TNF-α, and IL-17↓; liver LPO peroxidation, p53 and caspase-3 mRNA↓; serum IL-4↑; liver GSH content↑; liver GPx and SOD ↑	Antioxidant defense; Anti- inflammation; inhibition of apoptosis	Hassan et al. (152)

##### 4.2.6.1 Resveratrol (RES) and quercetin (QUR)

Al Humayed et al. (203) tested the protective effect of the combined polyphenolic compounds resveratrol (RES) and quercetin (QUR) in a rat model of liver cell ultrastructural damage induced by toxic doses of APAP. The results showed that transmission electron microscopy (TEM) images revealed marked changes in liver cell ultrastructure due to acute liver injury induced by excessive APAP, and these changes were significantly protected by RES + QUR. In addition, APAP significantly regulated TNF-*α*, IL-6, MDA, SOD, GPx, and ALT biomarkers, all of which were fully protected by RES + QUR. Therefore, RES + QUR effectively protects rats from APAP-induced acute liver injury, possibly through inhibiting inflammation and oxidative stress. In addition, APAP induces alterations to the glomerulus ultrastructure, which is protected by resveratrol plus quercetin, which also reduces blood levels of urea and creatinine, and biomarkers of oxidative stress such as MDA and inflammation such as TNF-α (204).

##### 4.2.6.2 Resveratrol (RES) and luteolin (LUT)

Based on the common use of resveratrol (RES) and luteolin (LUT), it can significantly enhance the bioavailability of quercetin and increase the systemic exposure to resveratrol. Combination therapy can also leverage their multi-component and multi-target characteristics.

Wu et al. studied the protective effects of combined resveratrol and luteolin against α-naphthyl isothiocyanate (ANIT)-induced cholestasis. Serum biochemical indicators in rats and liver tissue pathology indicated that the combined use of resveratrol and luteolin can improve liver function by inhibiting oxidative stress (antioxidant enzyme SOD and substrate GSH, and increasing the serum level of the lipid peroxidation product MDA). The levels of bile acids, deoxycholic acid, taurine conjugates, and glycine conjugates, as well as the ratio of taurine conjugates to their free forms, can serve as diagnostic indicators for cholestasis in rats. Furthermore, the combined use of resveratrol and luteolin can restore bile acid levels and demonstrate better protective effects compared to using either one alone. The above experimental studies suggest that the combined use of resveratrol and luteolin can protect rats from ANIT-induced cholestasis, with mechanisms closely related to the regulation of bile acid homeostasis and inhibition of oxidative stress (205).

##### 4.2.6.3 Astaxanthin (Asx) and capsaicin (cap)

The powerful antioxidants astaxanthin (Asx) and capsaicin (Cap) were co-encapsulated in liposomes, resulting in a synergistic antioxidant activity that was significantly higher than the summed activity of each antioxidant encapsulated individually. A study by Fukuta et al. used a carbon tetrachloride (CCl4) induced acute liver injury rat model, where the administration of CCl4 significantly increased the levels of aspartate aminotransferase (AST) and alanine aminotransferase (ALT). The combined intravenous administration of Asx-R encapsulated liposomes (Asx-R-Lipo) and Cap encapsulated liposomes (Cap-Lipo) significantly reduced the increase in AST and ALT levels caused by CCl4. Importantly, the treatment with Asx-R/Cap-Lipo exhibited a higher protective effect in acute liver injury compared to the combined treatment of Asx-R-Lipo and Cap-Lipo used separately. These results suggest that Asx-R and Cap co-encapsulated in liposome membranes can exert more effective antioxidant activity *in vivo*, and Asx-R/Cap-Lipo may become a promising antioxidant formulation for treating reactive oxygen species-related diseases (206).

##### 4.2.6.4 Navel orange peel extract, naringin and naringenin

The research by Osama et al. (155) investigated how the hydroethanolic extract of navel orange peel, along with naringin and naringenin, can prevent liver damage caused by APAP in male Wistar rats. The findings indicated that treating rats given APAP with these substances led to a notable reduction in elevated levels of serum AST, ALT, ALP, LDH, and GGT, as well as total bilirubin and TNF-*α*. Conversely, there was a significant increase in serum albumin and IL-4 levels. Additionally, the treatments decreased liver lipid peroxidation and increased liver GSH content, along with SOD, GST, and GPx activity, compared to the control group treated only with APAP. The peel extract was particularly effective in improving liver lipid peroxidation, GSH levels, and GPx activity. Furthermore, the treatments significantly reduced the levels of pro-apoptotic mediators p53, Bax, and caspase-3, while increasing the anti-apoptotic protein Bcl-2 in the rats treated with APAP. The treatments also improved liver histopathology, which was adversely affected by APAP, including issues like hepatocyte steatosis, cytoplasmic vacuolization, hydropic degeneration, and necrosis, alongside inflammation marked by the presence of mononuclear leukocytes and fibroblasts. In summary, the hydroethanolic extract of navel orange peel, along with naringin and naringenin, may help protect the liver in APAP-treated rats by enhancing antioxidant defenses and reducing inflammation and apoptosis.

In addition, the research by A et al. examined the protective effects of naringin, naringenin, and their combination against liver injury caused by Taxol (paclitaxel) in Wistar rats. Treatment with naringin and/or naringenin lowered the elevated serum levels of total bilirubin, AST, ALT, ALO, LDH, and ɣ-GT in rats treated with Taxol. It also significantly raised serum albumin levels, indicating liver improvement. The disrupted histological changes in the liver were notably improved with naringin and/or naringenin treatment in Taxol-treated rats. Additionally, these treatments reduced high hepatic lipid peroxidation and increased liver glutathione content, along with the activities of superoxide dismutase and glutathione peroxidase. Furthermore, the treatments lowered levels of alpha-fetoprotein and caspase-3, a pro-apoptotic mediator. The combination of naringin and naringenin appeared more effective in enhancing organ function and structural integrity. In conclusion, naringin and naringenin are suggested to offer hepatoprotective benefits by enhancing the body’s antioxidant defense, reducing inflammation, and inhibiting apoptosis (151).

##### 4.2.6.5 Naringin and hesperidin

The research by Hassan et al. evaluated the preventive effects of naringin and hesperidin, as well as their combination, on diclofenac-induced hepatotoxicity and the underlying mechanisms. Administration of naringin and hesperidin to mice injected with diclofenac significantly reduced serum levels of ALT, AST, LDH, ALP, GGT, total bilirubin, TNF-*α*, and IL-17, along with liver lipid peroxidation and the expression of liver p53 and caspase-3 mRNA. In contrast, serum IL-4 levels, liver GSH content, and the activities of liver GPx and SOD increased. Additionally, diclofenac-induced histological damage, including edema, cytoplasmic vacuolization, apoptosis, and focal necrosis of hepatocytes accompanied by inflammatory cell infiltration, showed significant improvement after treatment with naringin and hesperidin. In conclusion, naringin, hesperidin, and their combination (most effective) counteract diclofenac-induced liver injury through antioxidant, anti-inflammatory, and anti-apoptotic mechanisms (152).

Notably, before adding functional foods to the treatment plan for Drug-Induced Liver Injury (DILI), it’s crucial to carry out a thorough safety assessment. This involves studying the ingredients at a molecular level to understand their structure–activity relationships, dose–response relationships, mechanisms of action, and possible toxic effects. Treatment plans should be tailored to each patient’s specific situation, including their nutritional status, the degree of liver damage, and their personal preferences regarding functional foods. Furthermore, collaboration across disciplines such as nutrition, food science, and medicine is vital to ensure the effectiveness and safety of these foods. Educating patients about how to use functional foods properly, their potential benefits, and any possible side effects is an essential part of incorporating them into DILI treatment plans.

## 5 Conclusion and future perspective

DILI is very common and has attracted global attention. Over the past 20 years, edible natural products with potent hepatoprotective effects, including foods and food-derived bioactive compounds, have been studied. These compounds work by reducing oxidative stress, decreasing inflammation, maintaining normal mitochondrial function, inhibiting cell apoptosis, promoting autophagy, reducing hepatocyte necrosis, and repairing the structure and function of liver cells, providing promising alternatives for healthy dietary choices and the development of functional foods and drugs. Although most current evidence comes from animal experiments, many food-derived bioactive compounds have been confirmed to be effective hepatoprotective agents, and their mechanisms of action have been elucidated, necessitating additional clinical research. This article provides the latest information on the prevention and treatment of DILI with food and food-derived bioactive compounds. It offers guidance for physicians and nutritionists to advise people on consuming foods to protect against DILI and provides new insights for the development of new drugs for treating DILI.

Currently, although many food-derived compounds have shown promising results in preclinical studies, there is still a lack of clinical trials confirming their efficacy in treating DILI. To advance the management of DILI, well-designed randomized clinical trials are necessary to assess the effectiveness of food-derived compounds and the development of new molecules. Interdisciplinary collaboration between preclinical and clinical fields is an expedient and safe approach for accelerating the development of DILI treatment methods, reducing the risk of unexpected adverse events, and improving patient prognosis. In addition, before conducting randomized clinical trials, it is essential to use nanotechnology to enhance targeted drug delivery, control release, and improve solubility and bioavailability, as well as to carry out necessary pharmacokinetic, pharmacodynamic, and toxicological studies. Excitingly, experimental studies have been conducted to investigate the safety, tolerability, pharmacokinetics, and pharmacodynamics of purified (−)-EPI in healthy volunteers. The research indicates that no adverse reactions were observed in healthy volunteers using (−)-EPI, demonstrating high safety. Furthermore, it has also been found that the increase in NO metabolites, mitochondrial enzyme function, and plasma inhibitory hormone levels may be potential reasons for some beneficial effects of cocoa products or (−)-EPI reported in other studies (207). Furthermore, Fukuta et al. used liposome technology to co-encapsulate astaxanthin (Asx) and capsaicin (Cap) in liposomes, demonstrating higher protective effects in a rat model of acute liver injury induced by carbon tetrachloride (CCl4), pioneering the application of nanotechnology in combating drug-induced liver injury from food-derived compounds (206).
